# Establishing reliable neural health measures in adult cochlear implant users

**DOI:** 10.3389/fnins.2026.1890411

**Published:** 2026-08-13

**Authors:** Eleni Genitsaridi, Andrew Soulby, Efstratia Papoutselou, Patrick Boyle, Charlotte Garcia, François Guérit, Rachel Haines, Faizah Mushtaq, Cherith M. Campbell-Bell, Huib Versnel, Robert P. Carlyon, Douglas E. H. Hartley

**Affiliations:** 1 Rinri Therapeutics Ltd., Innovation Centre, Sheffield, United Kingdom; 2 St. Thomas’ Hearing Implant Centre, St. Thomas’ Hospital, London, United Kingdom; 3 National Institute for Health Research (NIHR) Nottingham Biomedical Research Centre (BRC), Nottingham, United Kingdom; 4 Hearing Sciences, Division of Clinical Neuroscience, School of Medicine, University of Nottingham, Nottingham, United Kingdom; 5 Advanced Bionics GmbH, European Research Center, Hannover, Germany; 6 Cambridge Hearing Group, MRC Cognition and Brain Sciences Unit, University of Cambridge, Cambridge, United Kingdom; 7 Department of Otorhinolaryngology and Head and Neck Surgery, UMC Utrecht Brain Center, University Medical Center Utrecht, Utrecht, Netherlands; 8 Nottingham University Hospitals National Health Service (NHS) Trust, Queen’s Medical Centre, Nottingham, United Kingdom

**Keywords:** auditory electrically evoked potentials, cochlear implant, longitudinal monitoring, neural health measures, outcome measures, psychophysics, reproducibility of results, spiral ganglion neurons

## Abstract

Following cochlear implantation, measures of auditory nerve function based on implant-driven electrical stimulation can be used to characterize neural health and support longitudinal monitoring and treatment outcome assessment, with particular relevance for advanced therapies targeting neural hearing loss. This study evaluated the reliability and measurement precision of neural-health measures derived from three complementary electrode-specific tests: panoramic electrically-evoked compound action potentials (PECAPs), interphase-gap (IPG) effects, and polarity effects. Six experienced adult cochlear implant users were assessed across five sessions by trained audiologists. PECAPs were measured across all electrodes, while IPG and polarity effects were assessed at three electrodes per participant. Test-retest reliability was quantified using intraclass correlation coefficients (ICCs), with measurements repeated over intervals ranging from 1 day to several weeks. Measurement error was further characterized using the minimum detectable change (MDC), defined as the smallest change required to exceed measurement error with 95% confidence. PECAPs neural-responsiveness measure and polarity effect on comfort levels demonstrated excellent reliability (ICC > 0.90), while IPG effects on ECAP amplitude growth function (AGF) slope showed good reliability (ICC > 0.80). Other variables showed moderate reliability, including polarity effect on threshold, and IPG effects on ECAP maximum amplitude, threshold, and AGF offset. MDC estimates indicated that relatively small within-electrode changes would be sufficient to exceed measurement error at the individual participant-electrode level. Exploratory analyses showed limited associations between measures, consistent with the idea that they may reflect distinct aspects of neural status. Together, these findings establish PECAPs, IPG effects and polarity effects as reproducible and complementary measures of auditory nerve health suitable for longitudinal assessment. Their combined use provides a robust physiological framework for monitoring neural status and detecting therapy-related changes in cochlear implant users, including in clinical studies of interventions targeting neural hearing loss. Further standardization of testing and analysis protocols, alongside expansion to larger multicentre cohorts will strengthen the generalizability and facilitate broader clinical and research adoption.

## 1 Introduction

In auditory research, the concept of neural health encompasses the structural integrity and functional status of spiral ganglion neurons (SGNs), reflecting the capacity of the auditory nerve to encode and transmit sensory information. Deficits in SGN function can degrade complex listening abilities such as speech-in-noise perception and sound localization (e.g., Zeng et al., 2005). SGN health can vary not only between individuals but also between ears of the same individual (e.g., Kaur et al., 2026; Seyyedi et al., 2014; Zimmermann et al., 1995). Moreover, neural degeneration can occur in distinct spatial patterns along a single cochlea with heterogeneous distribution of healthier and poorer segments, such as with predominant basal region deficits (Wu et al., 2023; Zimmermann et al., 1995).

Accurately measuring neural health is essential for a range of clinical applications, including diagnosis, prognostication, optimization of existing interventions, and treatment outcome assessment. Specifically, neural-health assessments can help distinguish sensory from neural hearing deficits (Genitsaridi et al., 2026; Liu et al., 2024; Rance and Starr, 2015). This distinction is crucial for selecting the most appropriate treatment pathway and for establishing realistic expectations about the potential benefits of interventions, including hearing devices. In addition, in cochlear implant (CI) users, knowledge of electrode-specific neural status can inform programming strategies, such as decisions on deactivating electrode contacts (henceforth referred to as electrodes), to improve performance (Pfingst et al., 2015). At the same time, as advanced therapies targeting restoration of SGN function emerge, measures of neural health are becoming increasingly important as outcome measures in clinical trials. In this context, such measures can provide evidence of target engagement and physiological change, complement functional outcomes, and enable more robust interpretation of treatment efficacy and inter-individual variability in response. In the longer term, reproducible neural-health measures may also support regulatory and reimbursement decision-making by helping to explain heterogeneity in clinical benefit and informing patient stratification. However, despite this growing need, there is currently no established test battery capable of reliably and sensitively quantifying changes in neural health.

CIs uniquely enable direct assessment of auditory neural function, making them powerful tools for advancing our understanding of neural health. Through intracochlear telemetry, clinicians and researchers can record electrophysiological responses that reflect the responsiveness and synchrony of SGNs. Utilizing electrode-level neural-health measures can enable capturing regional changes in SGN health and support interpretation of longitudinal changes. Establishing the reliability and sensitivity of these tests is a critical step toward their clinical application.

There is a range of objective and behavioral tests utilizing CIs proposed in the literature as potential measures of neural health, some used in clinical practice but most primarily in research settings. However, the underlying mechanisms that drive responses, as well as the relative strengths and limitations of these tests, are not yet fully understood. There is limited evidence from prospective longitudinal human studies of conditions that degrade neural health or that could improve it through therapeutic intervention. As such, available evidence comes mostly from animal studies, computational studies, or indirect indications from associations between different measures (Brochier et al., 2021a; Kalkman et al., 2022; Skidmore et al., 2022a).

The electrically-evoked compound action potential (ECAP) is widely used in clinical practice to objectively estimate the synchronized activity of auditory nerve fibers (Larsen et al., 2025). It is commonly used intraoperatively to assess neural responsiveness, while postoperatively it is used to confirm proper CI function, inform clinical programming decisions, and objectively measure auditory nerve responses over time. It has been demonstrated that daily ECAP measurements can be captured reliably through patient-operated home measurements, highlighting their feasibility and scalability for detecting longitudinal neural changes (Mushtaq et al., 2022; Mushtaq et al., 2024). Many variables can be derived from the ECAP response that may provide useful and complementary information on the underlying neural health, including the latency and threshold of response and amplitude-related variables such as the amplitude at maximum current and the slope of the amplitude growth function (AGF) (Larsen et al., 2025; Skidmore et al., 2022a). For example, a steeper ECAP slope has been suggested to associate with better neural health in various studies as discussed in Skidmore et al. (2022b).

Standard clinical ECAPs, however, can be influenced by non-neural factors, including electrode-modiolus distance, cochlear anatomy, and electrode impedance (Degen et al., 2020; İkiz Bozsoy et al., 2025; Kludt et al., 2025; Mlynski et al., 2021; Schvartz-Leyzac et al., 2020a; Schvartz-Leyzac et al., 2020b), and may reflect only part of the underlying neural physiology. Meaningful neural changes could thus be underestimated. More advanced approaches based on measuring differences in ECAP or behavioral responses when varying stimulation parameters can be used to complement ECAPs and contribute to a more comprehensive neural-health test battery (Brochier et al., 2021a). By choosing appropriate relative rather than absolute measurements, these methods can reduce the impact of non-neural factors so that the resulting estimates more accurately reflect underlying neural health (Brochier et al., 2021b).

An advanced approach for evaluating neural health through ECAP measurements is the panoramic ECAPs (PECAPs) method (Cosentino et al., 2016; Garcia et al., 2021). It utilizes the forward-masking artifact-reduction technique where masker and probe stimuli are used to obtain the ECAP response. The masker stimulus reduces the neural response to the probe stimulus due to the refractory period of the neurons. By comparing neural responses with and without the masker, the technique subtracts out artifacts from the probe, allowing for clear ECAP measurements. The PECAPs method evaluates ECAP responses at comfortable levels for masker and probe stimuli presented at all possible combinations of electrodes. It leverages the fact that ECAP amplitude reflects the overlapping excitatory areas of both masker and probe. These amplitudes are then processed by an algorithm that generates estimates for the width of current spread (sigma or σ value) and the synchronized neural responsiveness corresponding to neural health (eta or η value) for each electrode location. The η and σ values reflect the corresponding neural excitation at each location. These estimates may provide a more accurate reflection of neural activation patterns compared to standard ECAP measurements, particularly in cases of poor spatial selectivity, such as in the presence of neural dead regions (Cosentino et al., 2016). There is also evidence that PECAPs isolate the neural responsiveness from non-neural aspects of the electrode-neuron interface such as current spread and electrode-modiolus distance (Garcia et al., 2021; Garcia et al., 2024; Garcia et al., 2025).

The interphase-gap (IPG) effects approach is an advanced test that assesses changes in ECAP characteristics when the stimulus IPG is varied (Skidmore et al., 2022a). Studies have revealed that increasing the IPG makes neural stimulation more effective, demonstrated by electrophysiological and psychophysical response changes, including lower ECAP and behavioral thresholds, higher ECAP amplitudes and perceived loudness, and steeper ECAP AGF slopes (Carlyon et al., 2005; Hughes et al., 2018; McKay and Henshall, 2003; Prado-Guitierrez et al., 2006; Ramekers et al., 2014; Shepherd and Javel, 1999). These effects are likely associated with a reduction of the effect of the inhibitory (hyperpolarizing) phase on the excitatory (depolarizing) phase of biphasic stimuli when the two are further apart in time (McKay and Henshall, 2003; Zeng et al., 2004). Moreover, evidence from studies in both animals and humans suggest that changes in the effect of varying the IPG on ECAP-derived variables could be associated with differences in neural health (e.g., He et al., 2020; Prado-Guitierrez et al., 2006; Ramekers et al., 2022; Ramekers et al., 2015; Ramekers et al., 2014; Schvartz-Leyzac et al., 2020a; Schvartz-Leyzac et al., 2019; Schvartz-Leyzac and Pfingst, 2018; Zamaninezhad et al., 2023). Specifically, it is theorized that the increase in excitability observed when increasing the stimulus IPG is influenced by parameters affecting the integration time constant of the neural membrane (such as fiber diameter, myelination, and voltage-gated sodium channel kinetics) (Brochier et al., 2021a; Ramekers et al., 2022; Ramekers et al., 2014; Skidmore et al., 2022a). IPG-effect variables that have been investigated across studies, and for which there is at least some evidence of dependence on underlying neural health include ECAP latency, threshold, amplitude (at maximum delivered current or at AGF saturation), AGF slope, and offset (referring to the horizontal shift of the AGF) (e.g., Brochier et al., 2021a; Brochier et al., 2021b; He et al., 2020; Ramekers et al., 2022; Ramekers et al., 2015; Ramekers et al., 2014; Schvartz-Leyzac et al., 2019; Skidmore and He, 2021). However, based on existing literature the direction and degree of associations between IPG effects on the various ECAP characteristics and neural health are not straightforward. Moreover, some methodologies for retrieving variables of interest, such as the effect of the IPG on the slope of the ECAP AGF, can be more susceptible to non-neural factors and are not yet fully standardized (Brochier et al., 2021b; Skidmore et al., 2022b). Despite these limitations, IPG effects could provide valuable estimates of the underlying neural health.

In addition to objective electrophysiological responses, behavioral psychophysical measures can provide complementary insights into the auditory pathway function, capturing aspects of perception and neural encoding that are not always reflected in ECAP-based assessments. The lack of consistent correlation between objective and behavioral measures suggests they are influenced by different mechanisms along the auditory pathway, in addition to effects of between-measure differences in the stimuli (Thai-Van et al., 2004; Zimmerling and Hochmair, 2002). While ECAP thresholds primarily reflect the synchronized response of SGNs, behavioral responses may also be influenced by asynchronous peripheral activity and are additionally influenced by central processing mechanisms beyond the auditory periphery (He et al., 2024). Therefore, assessing both measures may provide a more comprehensive understanding of peripheral neural health. Although changes in standard behavioral measures, such as electric-stimulation thresholds, most comfortable loudness levels, and dynamic range, can indicate changes within the auditory system, more complex tests have been proposed to provide better estimates of neural health (Brochier et al., 2021a).

One such test, investigated by different research groups, is the polarity effects test (Carlyon et al., 2018; Jahn and Arenberg, 2019b), which assesses the effect of stimulus polarity (cathodic or anodic) on current levels corresponding to threshold and to comfortable sound perception. In humans, anodic-dominant stimuli are generally more effective at eliciting sound perception than cathodic-dominant, i.e., the current needed for the same loudness perception tends to be lower for anodic- than cathodic-dominant stimuli, particularly at comfortable levels (Carlyon et al., 2013; Guérit et al., 2018; Macherey et al., 2008; Macherey et al., 2006; Undurraga et al., 2013; Undurraga et al., 2010; van Wieringen et al., 2008). To avoid any platinum dissolution, charge needs to be balanced over a few milliseconds, and these polarity effects are therefore measured with asymmetric pulse shapes, which accentuate the perceptual dominance of one polarity while keeping charge balanced. All implant types can currently create asymmetric pulse shapes (Carlyon et al., 2013), with small differences in the types of asymmetries feasible (e.g., triphasic, quadra-phasic, pseudo-monophasic). The proposed value of polarity effects as a neural-health test is largely based on the theory that cathodic stimulation preferentially activates more peripheral locations of the auditory nerve, such as peripheral processes which can be lost first in some neurodegenerative aetiologies (Kaur et al., 2026; Ramekers et al., 2020; Spoendlin, 1975), whereas anodic stimulation preferentially activates more central locations (Brochier et al., 2021a; Carlyon et al., 2018; Recugnat, 2019). Thus, lower cathodic than anodic thresholds would suggest good neural health, whereas a shift to more effective anodic stimulation would be suggestive of compromised neural health. This aligns with studies showing that lower cathodic thresholds (corresponding to more negative polarity effects) were associated with lower focused behavioral thresholds and better performance in speech and spectro-temporal modulation detection tasks (Goehring et al., 2019; Jahn and Arenberg, 2019a; Mesnildrey et al., 2020). Consequently, a change in the polarity effect, specifically a reduction of the difference between cathodic and anodic thresholds, following a treatment aiming to improve neural health could provide strong evidence of treatment efficacy. However, it is worth noting that the relationship between polarity effects and underlying neural health is still under investigation and might not be straightforward; it might be determined by the exact mechanisms underlying neural dysfunction (e.g., related to loss or shortening of peripheral processes or other disorders such as changes in myelination or density of sodium channels), electrode array position (e.g., distance from the modiolus and insertion angle), and level and type of stimuli used (Kalkman et al., 2022; Konerding et al., 2025a; Macherey and Cazals, 2016).

The aim of this study was to establish the reliability of neural-health measures, specifically PECAPs, IPG effects, and polarity effects, that can support longitudinal assessment and serve as outcome measures in clinical trials of advanced therapies for neural hearing loss, such as the trial of a cell-based therapy, Rincell-1 (ClinicalTrials.gov: NCT07032038). Although several other candidate neural-health measures exist, these three were selected as they have been assessed in human studies by multiple independent research groups, their testing procedures can be standardized to be implementable in a clinical environment (e.g., by not requiring ECAP responses at saturation and allowing repeated longitudinal assessment with acceptable testing burden), they have distinct and complementary advantages and limitations, and are likely to capture distinct physiological processes relevant to SGN degeneration or regeneration.

The primary objective was to determine the test-retest reliability and minimum detectable change (MDC) for measures derived from these three tests in adult CI users with stable hearing, thereby establishing the degree of natural variability expected in a population where true changes in neural health are not anticipated. These benchmarks are essential for interpreting longitudinal changes during clinical trials in which treatment-related improvements are not yet quantified and may be electrode-specific. Secondary objectives were to informally assess the practical applicability and limitations of these methods when implemented by clinical audiologists in realistic clinical settings, and to explore the relationships among the three tests to understand whether they provide overlapping or complementary information about peripheral neural status.

## 2 Materials and methods

### 2.1 Participants

This study was approved by the Health Research Authority (HRA) and Health Care Research Wales (HCRW), with ethical approval granted by the East Midlands - Nottingham 2 Research Ethics Committee (REC reference 24/EM/0089), United Kingdom (UK). All participants provided written informed consent. Six adult users (≥18 years old) of Advanced Bionics CIs were recruited from a single UK centre (Guy’s & St. Thomas’ NHS Foundation Trust). Participants needed to have been implanted for at least a year prior to participation to allow measurements to stabilize post-cochlear implantation. Exclusion criteria included known cochlear abnormalities likely to affect measurements, and conditions that could change hearing function during participation, such as the use of ototoxic medications, or participating in other hearing-loss-related research. Additional eligibility criteria included the ability to read and understand English, normal or corrected-to-normal vision, and no major physical or mental health disorder or other factor that would hinder participation. There was an equal number of males and females, and the mean (and range of) age, age at implantation and duration of implantation were 51.3 (25.3–72.6), 46.0 (23.1–69.4), and 5.3 (2.1–10.2) years, respectively. Participant 1 had adult-acquired hearing loss with history of bilateral Ménière’s disease. All other participants had congenital or childhood-acquired hearing loss. Participant demographics and cochlear implantation characteristics, including the implanted device, are presented in Table 1. Participants were implanted with either the HiRes Ultra or HiRes 90k implants, both of which are equivalent in terms of electrical stimulation, with either the straight, HiFocus SlimJ electrode array, or the pre-curved HiFocus Mid-Scala electrode array. The latter is designed to sit more centrally within the scala tympani.

**TABLE 1:** Participant demographics and cochlear implantation history.

ID	Sex	Age (years)	Age at implantation (years)	Duration of implantation (years)	CI side	CI model	Number of active electrodes	Re-implanted
PT 1	Female	72.1	69.4	3.0	Left	HiRes Ultra Slim J	14	No
PT 2	Male	25.3	23.1	2.2	Right	HiRes Ultra Slim J	16	No
PT 3	Female	31.5	29.4	2.1	Left	HiRes Ultra Slim J	16	Yes
PT 4	Male	52.2	41.9	10.2	Right	HiRes 90K MS Advantage	16	No
PT 5	Male	57.8	48.0	9.9	Right	HiRes 90K MS advantage	16	No
PT 6	Female	68.9	64.3	4.6	Right	HiRes Ultra Slim J	14	Yes

For participants 3 and 6 (re-implanted), age at implantation and duration of implantation refer to the most recent implantation; initial implantation was approximately 4 and 3 years earlier for participants 3 and 6, respectively. CI, cochlear implant; ID, identification code; PT, participant.

### 2.2 Assessment procedures

PECAPs, IPG effects, and polarity effects were tested on five different days (sessions) for each participant. The interval between consecutive sessions was at least 1 day, with up to 63 days between the first and last session for any participant. All tests used research software [Bionic Ear Data Collection System (BEDCS), version 1.18] and research hardware [Platinum Series Speech Processor (PSP); Clinical Programming Interface (CPI) version II] provided by Advanced Bionics.

For PECAPs, custom MATLAB (MathWorks Inc.) scripts were used to control Advanced Bionics software and hardware. The paradigm followed the principles described in Garcia et al. (2021). In summary, the forward-masking artifact-reduction technique (original masker-probe paradigm; Abbas et al., 1999; Brown et al., 1990) was used to collect ECAP responses from every masker and probe electrode combination using cathodic-leading biphasic stimuli with approximately 20 pulses per second, 30-μs phase duration and 0-μs IPG presented at current levels determined based on participant comfort levels (see details below). Masker and probe levels were equal and the masker-probe interval was 400 μs. For each masker-probe combination, ECAPs were obtained from 50 averaged sweeps. ECAP responses were recorded from an electrode one or two positions from the probe electrode, keeping the stimulating-recording electrode separation as constant as possible. By default, the recording electrode was two positions apical to the probe, moved to two positions basal when the apical position would fall beyond the end of the array, and placed between the masker and probe electrodes when either default would otherwise coincide with the masker electrode.

IPG effects were tested on the BEDCS software. A forward-masking artifact-reduction technique (original masker-probe paradigm; Abbas et al., 1999; Brown et al., 1990) was used to collect ECAPs from biphasic stimuli with approximately 20 pulses per second, and both 0 and 30-μs IPG. Phase duration was 30 μs and both anodic- and cathodic-leading stimuli were assessed. The sampling rate was ∼56 kHz (every 18 μs). Each ECAP measurement was the average of 64 single-trace recordings. Each condition was assessed using masker stimuli at six current levels equally spaced from 250 μA up to a maximum level determined based on participant’s comfort (see details below). Probe stimuli levels were 10% below masker levels (calculated on a linear scale) and the masker-probe interval was 400 μs. ECAP responses were recorded from a fixed electrode one position apical to the stimulating electrode, so the stimulating-recording electrode distance was held constant across electrode locations and across both interphase-gap conditions.

Advanced Bionics implants have 16 independent current sources (one per electrode contact). The maximum voltage available to source current is 8 volts (nominal value; the actual supply voltage depends on the coupling between the headpiece coil and the implant coil). The voltage required to deliver a requested current depends not only on the current amplitude, but also on the electrode impedance and the stimulus phase duration. When the voltage required is below the maximum available, the current source is within compliance, and the requested current is delivered accurately. When the required voltage exceeds this limit (out-of-compliance condition), the actual delivered current is lower than requested. Because the BEDCS research software does not measure electrode impedance, requested currents were calculated manually using the electrode impedances, measured at the start of each session, and verified to be within compliance. In this manuscript, “compliance limit” refers to the maximum deliverable current for each stimulus condition, given the electrode contact impedance and specified phase duration. For calculating compliance limits, a more conservative supply voltage of 6.3 volts was used.

For both PECAPs and IPG effects, the maximum current level corresponding to comfortable perception was determined and used across sessions unless it needed to be reduced due to reaching compliance limits, or the presence of non-auditory sensations (NAS). For PECAPs, it involved a loudness scaling assessment at 5 electrodes to identify the “loud but comfortable” level (level 7 on the Advanced Bionics 11-point loudness scaling chart), which was used for stimulation. Levels for the remaining electrodes were estimated by linear interpolation. For IPG effects, three options for a maximum current level were pre-specified (750, 650, or 550 μA) for the masker stimulus. Stimuli with increasing current level were presented up to these maxima, and participants continuously rated comfort using the scaling chart. Presentation stopped when stimuli became “loud but comfortable.” The maximum presentation level that was comfortable across all electrodes was used for stimulation (same level across electrodes for each participant). Note that the stimuli used for comfort assessment were cathodic-leading with a 30-μs IPG. Since there was a possibility that stimuli of the remaining parameters assessed could be uncomfortable, participants were advised to let assessors know as soon as any stimulus was uncomfortable to stop presentation immediately, but that was never the case. For PECAPs, if any of the specified levels were above compliance limits, current levels were adjusted accordingly to be within limits while maintaining equal loudness across the array. Similarly, for IPG effects, a lower maximum current level was selected.

Polarity effects were assessed using custom MATLAB scripts controlling Advanced Bionics software and hardware. Stimuli were composed of anodic- or cathodic-leading pseudo-monophasic pulses at 480 pulses per second, with 0-μs IPG, 97-μs first phase duration, and second phase duration 8 times longer and amplitude 1/8 of the first phase (Macherey et al., 2006). Pseudo-monophasic pulses were used because their short, high-amplitude phases are likely more effective than their low-amplitude, long-duration phases (Macherey et al., 2006; Miller et al., 2001; Moon et al., 1993), producing larger polarity effects than biphasic pulses while preserving charge balance within a short time window to avoid platinum dissolution of the electrodes (Merrill et al., 2005). Testing procedures involved two main steps, loudness scaling and loudness balancing (both performed at every session). Procedures were similar to Macherey et al. (2006). For loudness scaling, the current levels corresponding to threshold, “comfortable but soft” (level 5), and “loud but comfortable” (level 7) perception for both anodic- and cathodic-leading stimuli were determined. Then, for loudness balancing, participants were presented with two 750-ms pulse trains (each composed of opposite-polarity pulses) with 300 ms in between them, and control was given to them to adjust the level of the second pulse train to match the loudness of the first. The first sound was always presented at level 5, and the starting level of the second sound was level 5 minus 4 dB. This loudness balancing step was repeated four times per electrode, with polarity of the initial stimulus being either cathodic- or anodic-leading (two times each).

For PECAPs, all 16 electrodes were assessed per session. For IPG and polarity effects, only three electrodes (usually e3, e8, and e13; see details for exceptions in results) were tested, because of time constraints.

In addition, electrode impedances were assessed twice per session per participant, at the beginning and the end of each testing session. Standard ECAP thresholds were assessed once at each of the five sessions using the Advanced Bionics Active Insertion Monitoring (AIM) system as described in previous studies (Mushtaq et al., 2022; Mushtaq et al., 2024). Speech perception was assessed once per participant (at a session chosen by the audiologist and participant) and included Arthur Boothroyd (AB) word perception tests in quiet and in noise and Bamford-Kowal-Bench (BKB) sentences perception in adaptive noise (Bench et al., 1979; Vickers et al., 2016). For both AB words in quiet and in noise, the signal was presented at an A-weighted sound pressure level (SPL) of 70 dB. For AB words in noise, speech-weighted noise at +10 dB signal-to-noise ratio (SNR) was used. The BKB sentences were presented at an overall fixed A-weighted SPL of 70 dB with the signal and noise levels adapted in opposing directions to find the SNR that corresponded to 50% correct sentence recognition (speech reception threshold; SRT). All tests were conducted by experienced CI audiologists who received specific training on performing the neural-health tests.

### 2.3 Data analysis

#### 2.3.1 Neural-health and other variables

PECAP “M” matrices were assembled from the ECAP amplitudes obtained for every combination of masker and probe electrode locations. For each ECAP waveform, the ECAP amplitude was determined automatically by identifying the first negative peak (N1) after the offset of the probe pulse, and the subsequent positive peak (P2). For participant 6, recording artifacts were present in the M matrix requiring 7% of the cells of M to be replaced with the value of the cell for opposite masker-probe combinations, and 29% to be linearly interpolated from neighboring cells. In all interpolation cases, at least 2 neighboring cells were artifact-free and used for interpolation. PECAPs data were analyzed using a custom MATLAB script to calculate the η and σ values per electrode as described in Garcia et al. (2021). PECAPs η can be presented both scaled by participant and rescaled across a group of participants (the latter allowing for across-participant comparison). Scaled η values (the output of the PECAPs algorithm) range from 0 to 1 and involve a scaling step relative to the maximum measured ECAP amplitude across electrodes for each testing session. These values represent the proportion of synchronous responsiveness of SGNs at a given electrode relative to all other electrodes in that participant’s array, where 0 indicates minimal synchronized neural responsiveness and 1 the highest possible responsiveness observed within that participant’s session.

For comparisons across participants and sessions this scaling was redone. The resulting values (rescaled, after removing within-participant and within-session scaling) represent the proportion of synchronous responsiveness of SGNs at a given electrode relative to the highest responsiveness across participants, sessions, and electrodes. To calculate rescaled η values, similar to Equation 11 in Garcia et al. (2021), the following transformation was applied to each value (Equation 1):


$$\mathrm{rescaled}\,\eta =\frac{\mathrm{maximum}\,\mathrm{amplitude}}{\mathrm{overall}\,\mathrm{maximum}\,\mathrm{amplitude}}*\mathrm{scaled}\,\eta$$ (1)

where the fraction numerator is the maximum ECAP amplitude for a testing session and the denominator is the overall maximum amplitude across sessions and participants. As such, rescaled η values also have a range from 0 to 1. Because this scaling is internal to each participant’s array, the absolute ECAP amplitude, which varies across CI users, does not need to be known or calibrated. Scaled η captures the relative distribution of neural responsiveness within a participant, whereas rescaled η enables comparison across users.

For PECAPs, an SNR was calculated as described in Garcia et al. (2021), Equation 12, using repeated ECAP measurements; SNR exceeded 10 dB for all sessions and participants (range 31.03–57.09 dB). The threshold of 10 dB was identified previously as the SNR above which the PECAP algorithm reliably estimates neural activation patterns (the combined contribution of neural responsiveness and current spread) with at least 90% accuracy. This was evaluated using a “backwards” PECAP model where the ground-truth neural activation patterns are known (Garcia et al., 2021).

For ECAPs measured during the IPG effects test, N1-P2 amplitudes were determined across all levels using a semi-automated approach, including manual selection of the peaks at the highest presentation level and automatic adjustment to nearby local extrema for the lower levels. The following variables were derived from the IPG effects test.

The IPG effect on amplitude at maximum stimulation current was calculated as the following ratio where Amp_30I*PG*_ is the amplitude (of N1-P2 peaks) at maximum current using stimuli with 30-μs IPG and Amp_0I*PG*_ the corresponding amplitude with 0-μs IPG measured in μV (Equation 2):


$$\mathrm{IPG}\,\mathrm{amplitude}=\frac{{\mathrm{Amp}}_{30\,I\,P\,G}-{\mathrm{Amp}}_{0\,I\,P\,G}}{{\mathrm{Amp}}_{30\,I\,P\,G}}$$ (2)

The IPG effect on slope of the AGF was calculated as the difference of the slope using 30-μs IPG (Slope_30I*PG*_) and the slope using 0-μs IPG (Slope_0I*PG*_) in μV/μA as follows (Equation 3):


$$\mathrm{IPG}\,\mathrm{slope}={\mathrm{Slope}}_{30\,I\,P\,G}-{\mathrm{Slope}}_{0\,I\,P\,G}$$ (3)

The AGF slope was calculated using the sliding-window method that looks for the steepest slope in the AGF curve as described in Skidmore et al. (2022b). Although ratio formulations can reduce the influence of non-neural factors (Brochier et al., 2021b), a ratio variable was not preferred for this measure as evidence linking ratio-based slope measures specifically to neural health is less robust (see section 4.3.2).

The IPG offset effect was based on quantifying the horizontal separation between the 0-μs and 30-μs IPG amplitude growth functions when both are expressed on a logarithmic input-output scale, following Brochier et al. (2021b). On these axes (ECAP amplitude in dB re 1 μV versus stimulus current in dB re 1 μA), the offset is the difference in stimulus current, at matched ECAP amplitude, between the 30-μs and 0-μs IPG functions, averaged across a supra-threshold band of the amplitude range. The current difference at each amplitude level was calculated as (Equation 4):


IPGoffset=x20*log10Lx30⁢I⁢P⁢G-20*log10Lx0⁢I⁢P⁢G (4)

where x indexes the ECAP amplitude levels sampled across the band spanning 70–90% of the amplitude range, with the range defined in dB from a nominal ECAP noise floor (10 μV) to the smaller of the maximum amplitudes reached by the two AGFs; Lx_30I*PG*_ and Lx_0I*PG*_ are the stimulus currents (μA) at which the 30-μs and 0-μs IPG AGFs respectively reach amplitude level x, obtained by linear interpolation on log-log input-output axes. At each amplitude level, the corresponding current was determined from the first point at which the AGF exceeded the noise floor, with any initial, non-monotonic portion excluded. The offset was averaged over levels on a 0.1-dB grid across this band. Restricting the average to the 70–90% band, which lies on the near-linear, supra-noise portion of both functions while remaining below saturation, follows the rationale of Brochier et al. (2021b) on comparing the linear portions of the log-transformed AGFs, while allowing for an automatic selection of regions. An example of this procedure is presented in Supplementary Figure 1.

The IPG effect on ECAP threshold was calculated as the log-ratio (in dB) of the threshold using 30-μs IPG to the threshold using 0-μs IPG, a difference of the same form as Equation 4. The ECAP threshold was defined as the intercept of the AGF slope with the x-axis. All IPG-effect variables were assessed using both anodic- and cathodic-leading stimuli. For each of these conditions, ECAP waveforms were quality-checked to exclude unreliable measurements due to lack of growth of amplitude. This required that, for data from a participant-electrode unit (i.e., one participant-electrode combination) to be included, N1-P2 amplitudes at the three highest stimulation levels for both 0- and 30-μs IPG stimuli demonstrated close-to-monotonic growth (or plateau) in two or more sessions. All included participant-electrode units also reached a maximum N1-P2 amplitude above 80 μV at the highest assessed level, well above typical ECAP noise floors of approximately 5–20 μV (Schvartz-Leyzac and Pfingst, 2016), ensuring a substantial signal margin relative to the measurement noise.

Comparison of IPG-effect variables across sessions also requires a consistent maximum stimulation current, because the IPG effect on amplitude at maximum current (Equation 2) and the IPG offset effect (Equation 4) are referenced to the maximum stimulation level. Moreover, although the slope and threshold estimates are relatively insensitive to small variations in stimulus level, estimates obtained at materially different maximum levels, particularly when saturation is not reached, may not be comparable; such measurements (for example following reductions in compliance limits or the presence of NAS) were therefore treated as non-comparable (Table 2).

**TABLE 2:** Overview of non-available data.

Test	Non-available data	Main reasons
PECAPs	∼13%	∙ Non-existent due to deactivated electrodes. ∙ Non-comparable resulting from sessions using different current levels (due to reductions in compliance limits or presence of NAS).
IPG effects	∼30%	∙ Non-existent due to insufficient growth of amplitude at increasing currents. ∙ Non-comparable resulting from sessions using different current levels (due to reductions in compliance limits).
Polarity effects	∼8%	∙ Missing due to procedural issues (e.g., misinterpretation of instructions, time constraints). ∙ Non-existent resulting from testing not completed due to NAS.

NAS, non-auditory sensations.

For polarity effects, two variables were derived. The polarity effect on threshold was calculated as the difference in cathodic- and anodic-leading stimuli current (expressed in dB) at threshold of perception using the following formula (Equation 5):


$$\mathrm{polarity}\,\mathrm{effect}\,\mathrm{on}\,\mathrm{threshold}=20*\log_{10}\,\frac{\mathrm{cathodic}\,\mathrm{current}\,\mathrm{in}\,\mu \,A}{\mathrm{anodic}\,\mathrm{current}\,\mathrm{in}\,\mu \,A}$$ (5)

The polarity effect on comfort was calculated as the difference in cathodic- and anodic-leading stimuli current at comfortable levels measured in dB (as in Equation 5) for each repeat of the loudness balancing experiment and then averaged across the four repeats.

In addition to variables from the main three tests, standard ECAP thresholds and speech perception variables were also used in the clustering analysis. Standard ECAP thresholds were measured in Advanced Bionics clinical units (cu) and determined automatically by the AIM system. If a threshold was not detected, values were imputed with the maximum stimulation level used (250 cu). Speech perception variables included BKB sentences (dB SNR), AB word scores in quiet (% correct words), and AB word scores in noise (% correct words).

#### 2.3.2 Intraclass correlation coefficient

Reliability of repeated measurements was assessed using the intraclass correlation coefficient (ICC). It assesses the degree of agreement among repeated measurements within defined groups, quantifying how much of the total variability in the data is attributable to differences between groups rather than within them. ICC was estimated using a one-way random-effects model, corresponding to ICC(1,1) in the Shrout and Fleiss (1979) framework, fit via the “performance” package’s icc() function, which extracts variance components from an lme4 mixed-effects model in R (Bates et al., 2015; Lüdecke et al., 2021). This model-based approach is robust to missing data and unbalanced group sizes. ICC(1,1) was used because sessions were scheduled per participant and did not correspond to common occasions across the cohort. Here, each ICC group corresponded to one participant-electrode unit, reflecting the assumption that test-retest measurements within each unit should be consistent. ICC was calculated as (Equation 6):


$$I\,C\,C=\frac{s_{g\,r\,o\,u\,p}^{2}}{\left(s_{g\,r\,o\,u\,p}^{2}+s_{r\,e\,s\,i\,d\,u\,a\,l}^{2}\right)}$$ (6)

where s^2^_*group*_ represents the variance between participant-electrode groups, and s^2^_*residual*_ represents the residual (within-group) variance. ICC ranges from 0 to 1, with higher values indicating greater within-group consistency. Following Koo and Li (2016), ICC values can be interpreted as indicating poor reliability when < 0.50, moderate reliability when 0.50–0.75, good reliability when 0.75–0.90, and excellent reliability when > 0.90. An important limitation, which must be considered when interpreting results, is that ICC depends on sample heterogeneity. Specifically, large between-group variance can inflate ICC even when within-group consistency is modest, and conversely, low between-group variance can produce a low ICC even when within-group consistency is high.

#### 2.3.3 Minimum detectable change

To complement ICC, the MDC was calculated to quantify the smallest change between two measurements of an individual participant-electrode unit that can be interpreted as exceeding measurement error (Portney, 2020; Weir, 2005). The MDC is particularly relevant in clinical trials, where distinguishing real treatment effects from random variation is essential. The MDC was derived from the standard error of measurement (SEM) (Equation 7):


$$S\,E\,M=S\,D*\sqrt{1-I\,C\,C}$$ (7)

where SD is the standard deviation of all observations across participant-electrode units and sessions for that variable. The MDC_95_ (the MDC at the 95% confidence level) was then computed as (Equation 8):


$$M\,D\,C_{95}=1.96*\sqrt{2}*\text{SEM}$$ (8)

Because MDC95 depends on both ICC and SD, variables with high SD relative to within-unit variability can yield large MDC95 values even when ICC is high, and vice versa.

#### 2.3.4 Associations across tests

Although the study was not powered to detect associations between tests, an exploratory *post-hoc* analysis was conducted, given the limited literature on how neural-health measures relate to one another. To limit the number of comparisons given the small sample size, the analysis was restricted to variables with the good or excellent test-retest reliability. For each participant-electrode unit, mean values across sessions were used per variable. For each variable pair, units missing values for either variable were excluded from both variables. To focus on within-participant patterns and remove any effects due to between-participant variability, values for each test were centered within participants (subtracting each participant’s mean across electrodes). Correlations were assessed using Pearson’s coefficients per variable pair (skewness of distributions ranged from -0.43 to 0.55 calculated using the “e1071” R package; Meyer et al., 2024). To evaluate statistical significance, the degrees of freedom were adjusted for within-participant centring by deducting one degree of freedom per participant, following the convention for repeated-measures correlations (Bakdash and Marusich, 2017; Bland and Altman, 1995). For each correlation, 95% confidence intervals were computed using Fisher’s z-transformation, with the standard error adjusted to reflect the reduced degrees of freedom. Given the exploratory nature of the analysis and the number of variable pairs assessed, a stricter significance threshold of α = 0.01 was adopted in place of formal multiple-comparison correction.

To further explore relationships among variables and across participants, a heatmap was generated with variables in columns and participants in rows. As above, only variables with good or excellent reliability from the three neural-health tests were included (using session-mean values). In addition, standard ECAP thresholds and the three speech perception variables were included. All variables were standardized to have zero mean and unit SD, using measurements from electrodes e3, e8, and e13 only. For the non-speech-related variables, values were then averaged across electrodes to obtain a single estimate per participant. To ensure that all variables in the heatmap encode neural health in the same direction, values for variables for which lower values are thought to indicate better neural health (BKB SNR, polarity effect on comfort, and standard ECAP thresholds) were multiplied by -1. Hierarchical clustering using average linkage and Euclidean distance was applied to standardized data to order participants and variables by similarity, and the results were visualized using a heatmap (Kolde, 2019). For visualization purposes only, values were additionally min-max scaled to [0, 1] within each variable.

All data processing and analyses were performed using MATLAB 2023b, Python 3.9 and R 4.4.1. Additional R packages used included “ggplot2,” “hrbrthemes,” “patchwork,” “ragg,” and “viridis” (Garnier et al., 2024; Pedersen, 2025; Pedersen and Shemanarev, 2025; Rudis, 2024; Wickham, 2016).

## 3 Results

### 3.1 Clinical observations and descriptive statistics

Appropriately trained clinical audiologists were able to perform all tests independently. Mean testing duration per session was 47 min for PECAPs, 29 min for IPG effects, and 47 min for polarity effects (averaged across participants and sessions). For PECAPs and IPG effects, loudness scaling was performed only at the first session, and this additional duration was included when calculating the mean across sessions. Descriptive statistics for all variables assessed in this study are provided in Supplementary Table 1. Non-available data were classified as missing (testing not completed), non-existent (e.g., deactivated electrodes), or not meaningful (e.g., non-comparable measurements recorded at different current levels). Approximately 13% of PECAPs data, 30% of IPG effects data, and 8% of polarity effects data were non-available (main reasons summarized in Table 2). The higher non-availability for IPG effects primarily reflects the quality-check criterion requiring monotonic AGF growth (section 2.3.1), which excluded participant-electrode units with insufficient growth at one or both IPG values. Some of the non-existent and non-comparable data were related to the presence of NAS, reported during PECAPs testing by participant 5, and during polarity effects testing by participants 5 and 6. NAS occurred across a wide range of current levels including high levels that remained comfortable (e.g., 201–420 μA for participant 5 during polarity effects testing). All instances of NAS were managed per local clinical practice, with data collection deprioritised relative to the participants’ safety and comfort (e.g., by testing different electrodes for polarity effects or at lower levels for PECAPs). For polarity effects, for participant 6 sessions 3 and 5, e12 was tested instead of e13, because the participant reported NAS for e13. Data from e12 were used in test-retest reliability analysis (tested in three sessions) and from e13 in association analyses (tested in one session but corresponding to electrode tested for IPG effects). Impedance values per participant before and after each testing session can be found in Supplementary Figure 2.

### 3.2 Test-retest reliability and minimum detectable change

**FIGURE 1:**
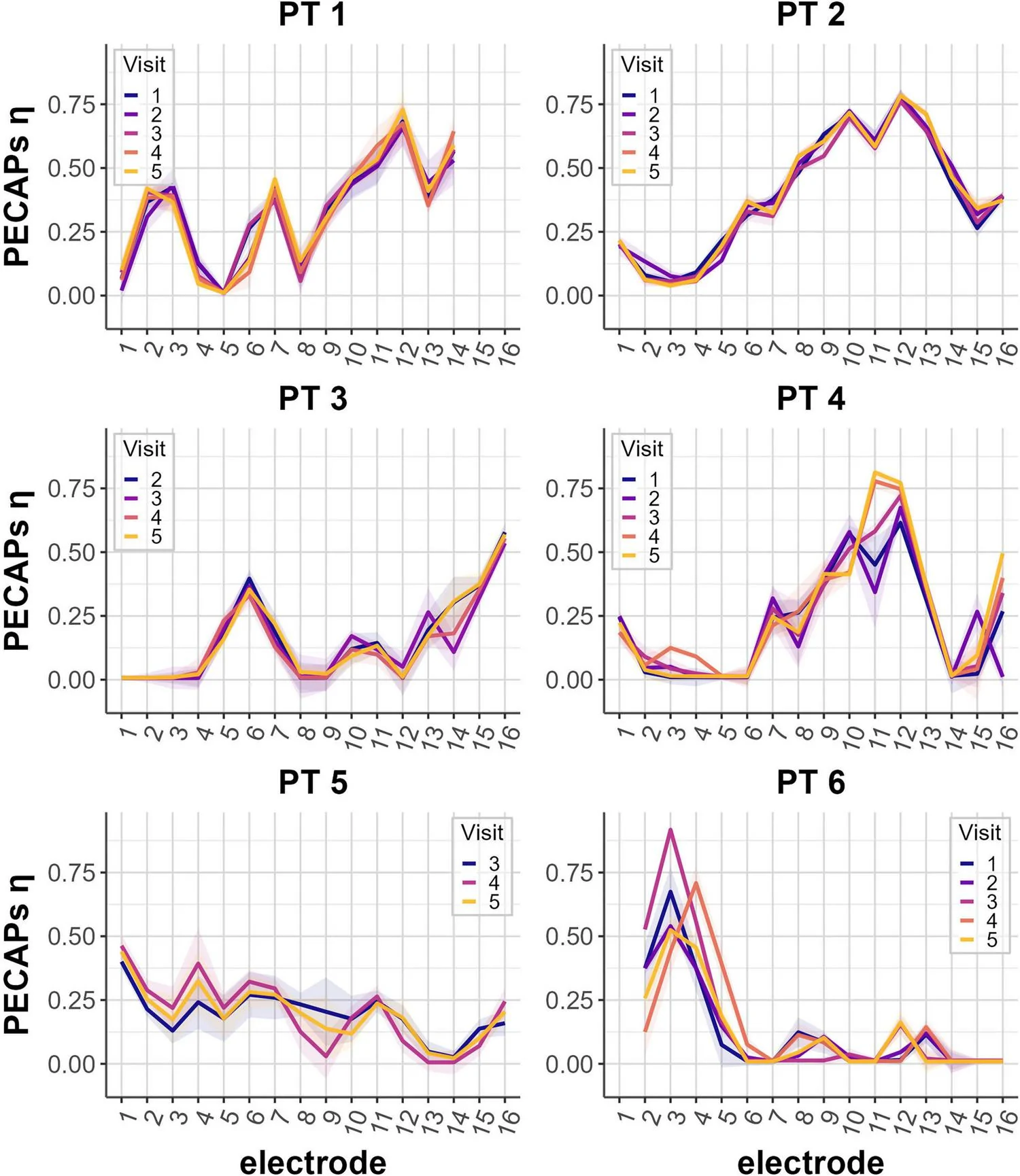
PECAPs η (scaled values) across electrodes and sessions for each participant showing inter- (colored lines) and intra-session (shaded areas) variability. PT: participant.

**FIGURE 2:**
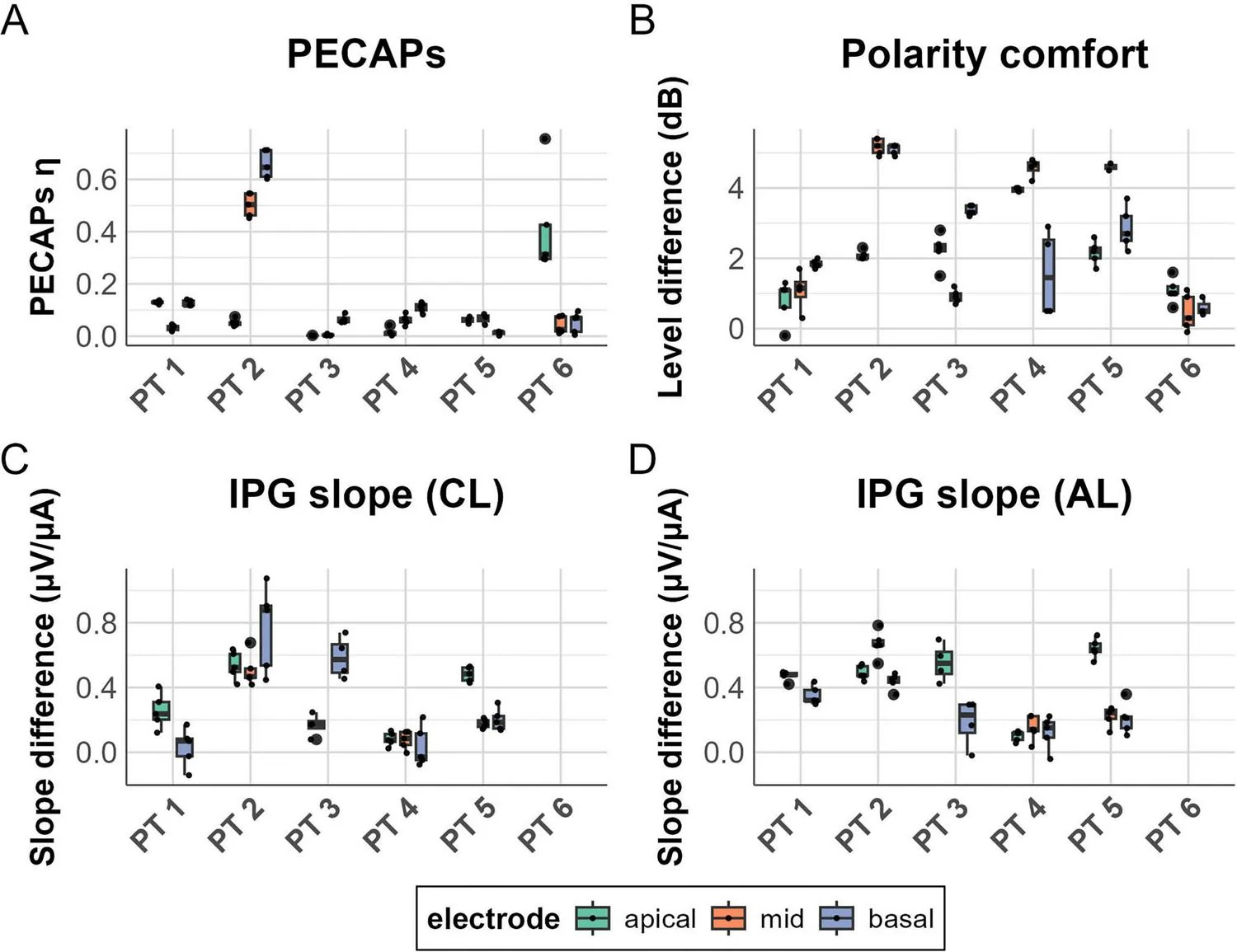
Boxplots of repeated measurements by participant-electrode unit for **(A)** PECAPs rescaled η values (only for e3, e8, and e13), **(B)** Polarity effect on comfort, **(C)** IPG effect on slope (cathodic-leading), **(D)** IPG effect on slope (anodic-leading). IPG effects data from middle electrodes for participants 1 and 3 and from all electrodes from participant 6 have been excluded due to insufficient amplitude growth at increasing levels. Apical (green; left), mid (orange; middle), and basal (blue; right) electrodes per participant correspond to e3, e8, and e13 (except for polarity comfort for participant 6 for whom the basal electrode is e12). AL, anodic-leading; CL, cathodic-leading; PT, participant.

Variables from all three tests demonstrated high test-retest reliability (Table 3). Excellent reliability was observed for PECAPs η values and polarity effect on comfort (ICC > 0.90), while good reliability was observed for IPG effect on slope (ICC ≥ 0.8; Table 3). Across these variables, results for individual participant-electrode units were generally consistent, with PECAPs η values largely overlapping across sessions for each electrode (Figure 1) and measurements showing low within-unit dispersion and high between-unit separation (Figure 2). Across-session SDs within participant-electrode units (Table 3) ranged from 0.000 to 0.197 (mean = 0.039) for rescaled PECAPs η; 0.030–0.149 μV/μA (mean = 0.079) for IPG effect on slope (anodic-leading); and 0.058–1.258 dB (mean = 0.447) for polarity effect on comfort. Higher variability can be observed for some individual units (e.g., participant 4, electrode e13, for polarity effect on comfort; Figure 2). Results from estimated MDC at the 95% confidence level for all variables are also presented in Table 3.

**TABLE 3:** Test-retest reliability and MDC results for selected variables.

Variable	Mean	Within participant-electrode			Across participant-electrode			ICC (CI _95_)	MDC_95_
		Variance	SD (min–max SD)	Max Range	Variance	SD	Range		
PECAPs η (rescaled)	0.137	0.002	0.039 (0.000–0.197)	0.461	0.029	0.171	0.783	0.95 (0.92, 0.96)	0.112
PECAPs η (scaled)	0.247	0.003	0.058 (0.000–0.203)	0.485	0.046	0.215	0.912	0.93 (0.90, 0.95)	0.164
IPG amplitude (CL)	0.322	0.003	0.056 (0.005–0.096)	0.235	0.006	0.078	0.410	0.66 (0.39, 0.80)	0.151
IPG amplitude (AL)	0.346	0.003	0.056 (0.010–0.137)	0.305	0.008	0.087	0.366	0.70 (0.45, 0.83)	0.153
IPG slope (CL) (μV/μA)	0.298	0.013	0.113 (0.028–0.265)	0.627	0.055	0.235	1.216	0.82 (0.64, 0.90)	0.304
IPG slope (AL) (μV/μA)	0.354	0.006	0.079 (0.030–0.149)	0.315	0.038	0.194	0.826	0.86 (0.72, 0.93)	0.212
IPG offset (CL) (dB)	-1.953	0.131	0.361 (0.060–0.692)	1.749	0.160	0.400	2.826	0.52 (0.24, 0.71)	0.988
IPG offset (AL) (dB)	-1.869	0.050	0.224 (0.021–0.426)	0.979	0.039	0.197	1.562	0.39 (0.11, 0.59)	0.612
IPG threshold (CL) (dB)	-1.372	0.395	0.629 (0.148–1.633)	3.386	0.950	0.974	4.905	0.70 (0.44, 0.83)	1.719
IPG threshold (AL) (dB)	-1.292	0.318	0.564 (0.126–1.073)	2.670	0.515	0.718	4.497	0.61 (0.33, 0.77)	1.532
Polarity threshold (dB)	1.063	1.487	1.219 (0.253–2.759)	6.765	2.056	1.434	10.242	0.55 (0.31, 0.72)	3.358
Polarity comfort (dB)	2.420	0.200	0.447 (0.058–1.258)	2.400	2.377	1.542	5.600	0.93 (0.87, 0.96)	1.193

For PECAPs, data from all electrodes are included. For polarity effects for participant 6, data from e12 (measured three times) and not e13 (measured once) are included. The mean value corresponds to the overall mean across participant, electrodes, and sessions. Within participant-electrode variance and SD correspond to the mean values across units. For SD the minimum and maximum SD values across units are presented in parenthesis. For ICCs the 95% confidence intervals are given in parenthesis. AL, anodic-leading; CL, cathodic-leading; CI_95_, 95% confidence intervals; ICC, intraclass correlation coefficient; MDC_95_, Minimum Detectable Change at 95% confidence level; SD, standard deviation.

**FIGURE 3:**
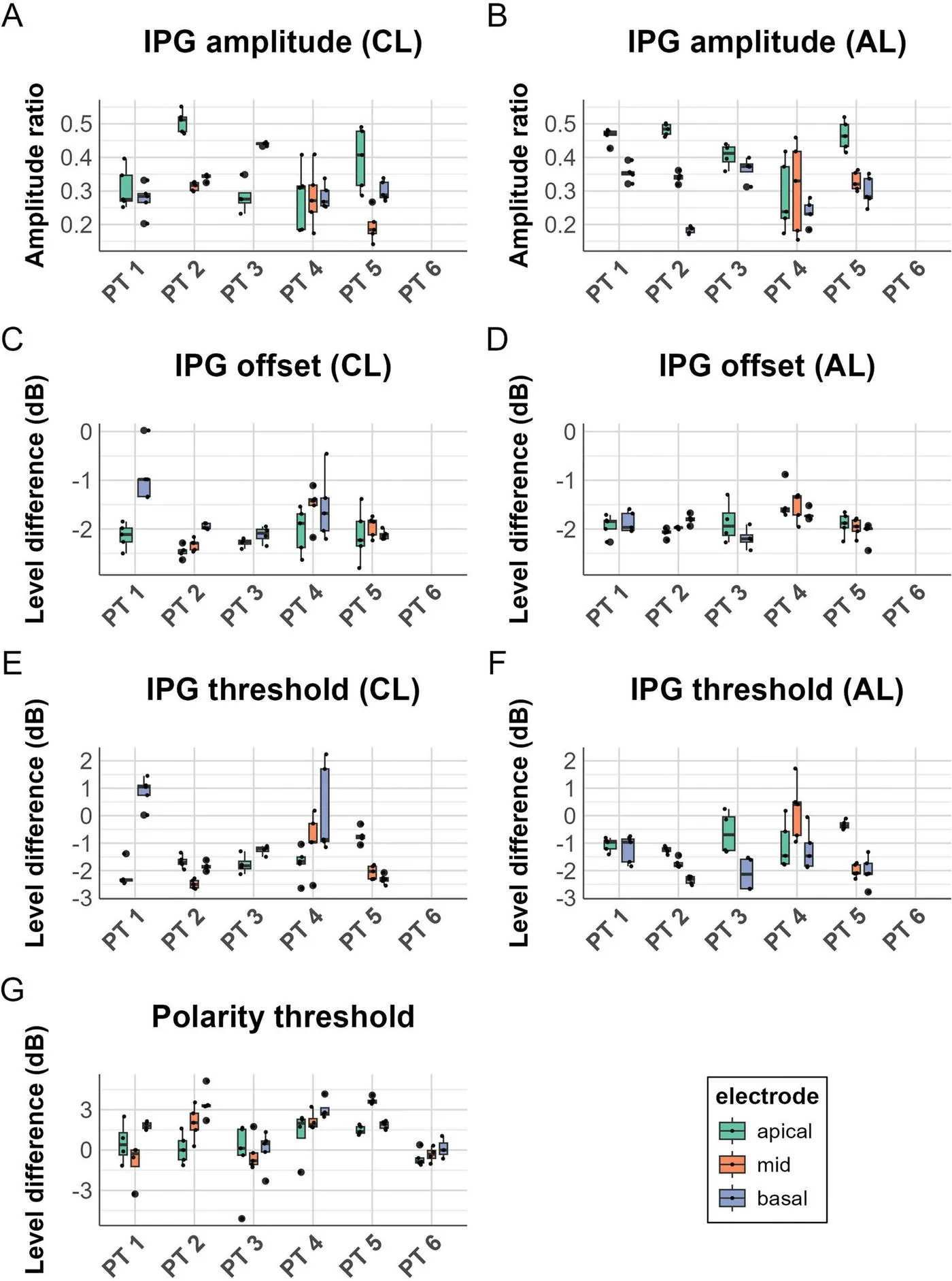
Boxplots of repeated measurements by participant-electrode unit for **(A)** IPG effect on amplitude (cathodic-leading), **(B)** IPG effect on amplitude (anodic-leading) **(C)** IPG offset effect (cathodic-leading), **(D)** IPG offset effect (anodic-leading), **(E)** IPG effect on threshold (cathodic-leading), **(F)** IPG effect on threshold (anodic-leading), **(G)** polarity effect on threshold. IPG effects data from middle electrodes for participants 1 and 3 and from all electrodes from participant 6 have been excluded due to insufficient amplitude growth at increasing levels. Apical (green; left), mid (orange; middle), and basal (blue; right) electrodes per participant correspond to e3, e8, and e13 (except for polarity threshold for participant 6 for whom the basal electrode is e12). AL, anodic-leading; CL, cathodic-leading; PT, participant.

Polarity effect on threshold, and IPG effects on amplitude, threshold and offset (cathodic-leading stimuli only) showed moderate reliability with ICC ranging from 0.52 to 0.70 (Figure 3 and Table 3). IPG offset effect from anodic-leading stimuli had lower ICC (0.39), but this reflected lower between-unit variance for anodic-leading stimuli (across-unit SD = 0.197) compared to cathodic-leading stimuli (across-unit SD = 0.400), rather than poor within-unit consistency (within-unit SD = 0.224 vs. 0.361, respectively). This is also reflected in the lower MDC_95_ value of IPG offset effect for anodic- compared to cathodic-leading stimuli (0.612 vs. 0.988 dB, respectively), indicating that smaller within-unit changes are detectable for the anodic-leading variable despite its lower ICC. Overall, no consistent superiority of one polarity over the other was observed across IPG-effect variables.

### 3.3 Test associations

**FIGURE 4:**
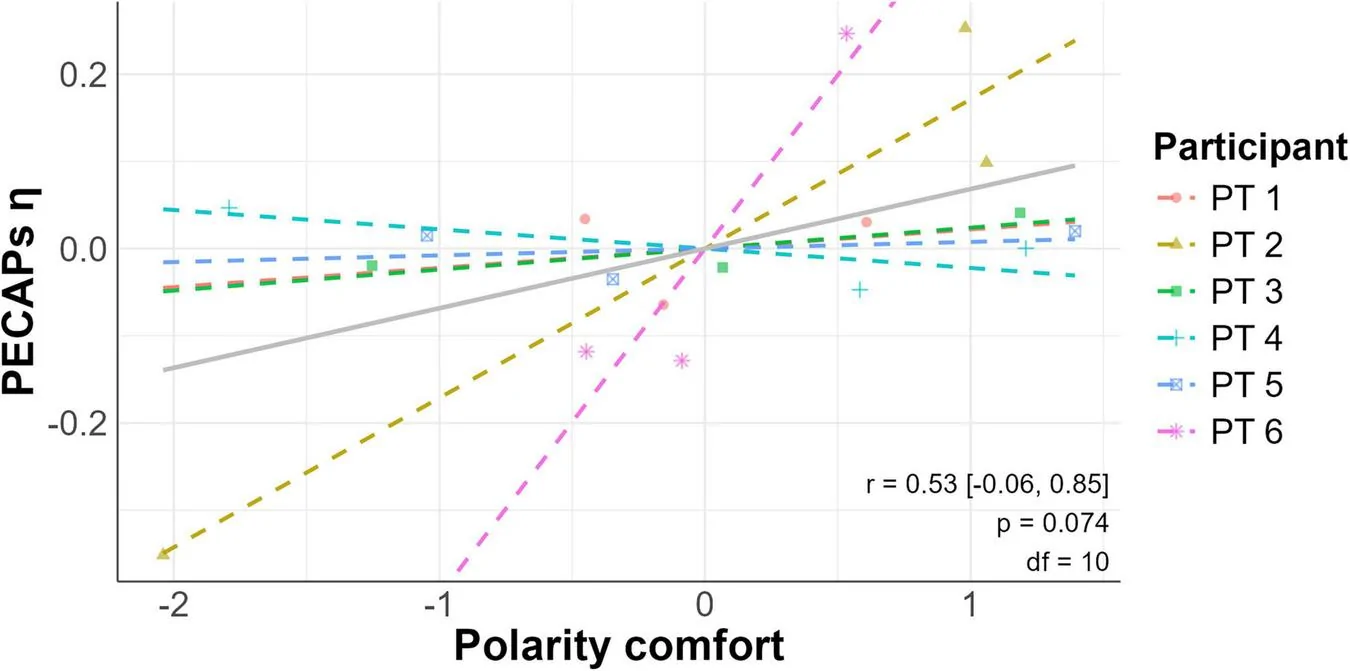
Pairwise associations between PECAPs η (rescaled estimates) and polarity effect on comfort. Data are centered for each participant to focus on within-participant patterns. Pearson’s correlation coefficient (r) with 95% confidence intervals in brackets, *p*-value (*p*), and degrees of freedom (df) are shown on the bottom right corner. For visualization, linear regressions lines are shown (colored per participant; gray for overall dataset).

To explore relationships between neural-health estimates from different tests, two analyses were performed. First, for pairs of variables, within-participant correlations were investigated after centring data within each participant to remove between-participant differences. Five pairs were assessed including PECAPs η (rescaled) and polarity effect on comfort, PECAPs η and IPG effect on slope (anodic- and cathodic-leading), and polarity effect on comfort and the same IPG-effect variables. No statistically significant correlations were observed across these analyses (all *p* > 0.05). Figure 4 presents an example of these pair-wise associations (between PECAPs η and polarity effect on comfort).

**FIGURE 5:**
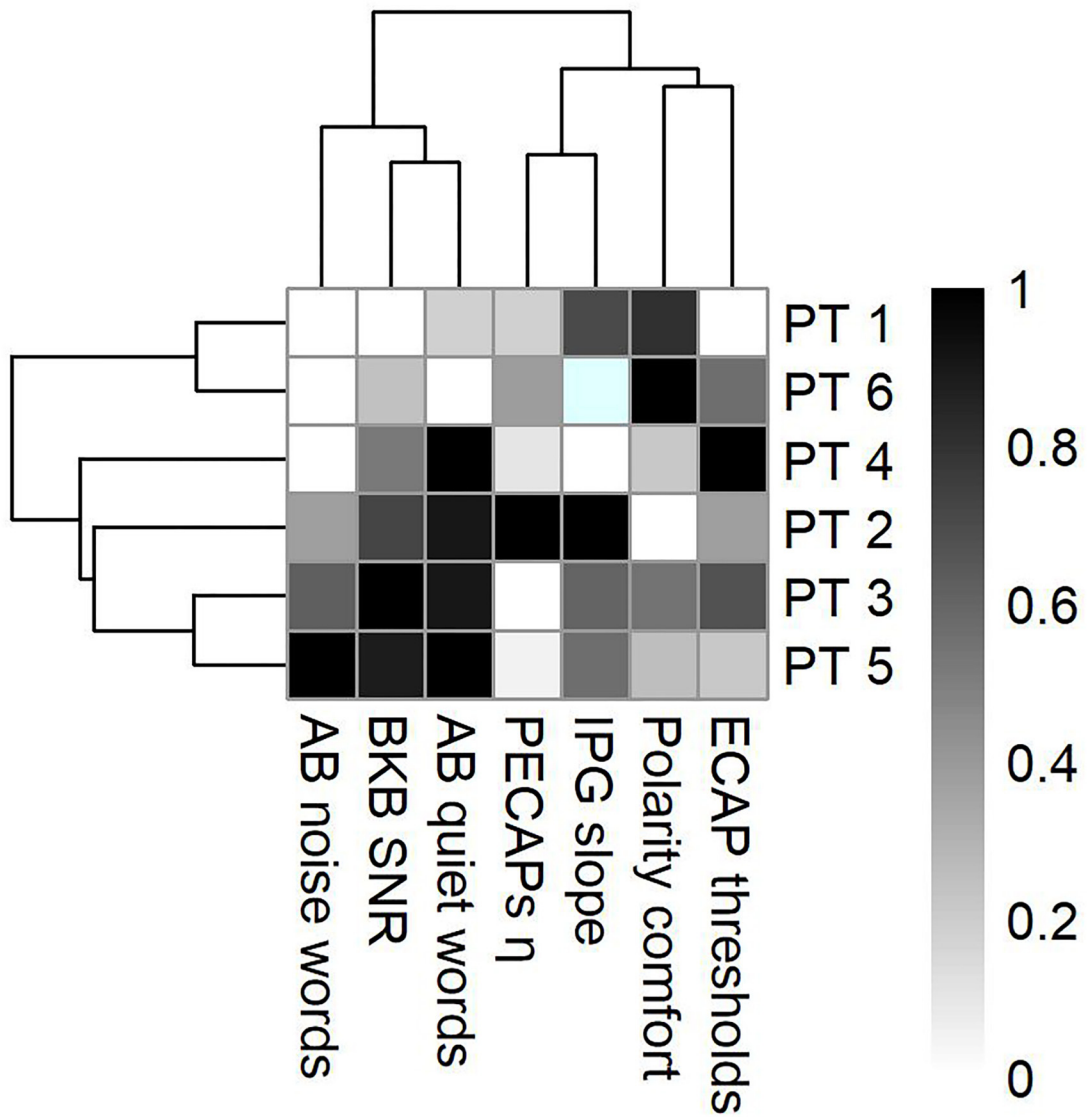
Heatmap of neural-health estimates (columns) across participants (rows) created using hierarchical clustering. Mean values across electrodes were used where applicable. For PECAPs η, rescaled values from e3, e8, and e13 were included. Before clustering, all values were scaled with mean 0 and standard deviation of 1 and reversed as needed so that higher values correspond to expected better neural health. For visualization, values where further scaled to zero-to-one range, so that for each variable one (black) corresponds to the highest estimated and zero (white) to the lowest neural health. Dendrograms (top: variables; left: participants) show how clusters merge at various distances; the height of each merge represents the dissimilarity between clusters with shorter heights indicating higher similarity. Values for IPG effects for participant 6 were not available (cyan). Results from anodic-leading stimuli are used for IPG-effect variables.

Second, a heatmap visualization was used to descriptively illustrate patterns of estimates across tests (Figure 5). The heatmap shows that variables from the three neural-health tests do not vary in the same way across participants, as can be seen in the patterns within each row. For example, for participant 2 average PECAPs η suggested relatively good neural health whereas polarity effect on comfort suggested poor neural health. Inspection of the heatmap further suggests that estimates from PECAPs, IPG effects, and polarity effects do not always align with speech perception or standard ECAP thresholds. Per-unit session means for variables used in this analysis are provided in Supplementary Table 2.

## 4 Discussion

### 4.1 Test-retest reliability

Results of this study showed that PECAPs, IPG effects, and polarity effects can provide estimates of neural health with high test-retest reliability. Despite their research-stage status, good test-retest reliability was demonstrated, with at least one variable per test having ICC ≥ 0.86 (Table 3). Observed ICCs are comparable to ICCs reported for other electrophysiological measures used in clinical audiology settings, such as auditory brainstem response (ABR) wave amplitudes and latencies, envelope following response (EFR) amplitudes, and middle ear muscle reflex (MEMR) thresholds (ICCs ranging from approximately 0.80 to 0.93), and substantially higher than for summating potential measures (ICCs ranging from approximately 0.18 to 0.47) (Guest et al., 2019; Prendergast et al., 2018).

For individual participant-electrode units (Figure 2), some showed substantially higher across-session variability, though not consistently across variables. Participant 4 showed notably high variability for some electrodes across several variables. For polarity effect on comfort, variability was higher for the basal electrode (e13); for PECAPs η, for e11 and e15; and for IPG-effect variables variability was often high across electrodes. Although this study was not designed to evaluate how individual clinical characteristics affect test variability, this pattern suggests that individual factors, possibly including pathophysiological characteristics, may influence stability, and warrants further investigation in larger samples. Despite the presence of such individual cases with higher variability, the aggregate reliability across the cohort remained high (section 3.2; Table 3).

### 4.2 Minimum detectable change and minimal clinically important difference

The Minimal Clinically Important Difference (MCID) represents the smallest change in an outcome considered meaningful by patients or clinicians. Establishing an MCID requires a direct linkage between measured change and clinical or functional benefit, typically via patient-reported outcomes, functional anchors, or robust associations with clinically relevant endpoints. At present, MCIDs have not been established for the neural-health measures evaluated in this study. The MDC provides a statistically grounded threshold indicating the smallest change exceeding measurement error. Changes below the MDC cannot be reliably distinguished from noise, whereas changes exceeding it are candidates for clinical meaningfulness, pending further validation. MDC values apply at the individual participant-electrode level. For multiple-electrode or group-level interpretation, the proportion of electrodes for which changes exceed the MDC, can complement group-level summary statistics (Portney, 2020).

Variables from all three tests showed MDC values indicating that relatively small within-electrode changes would suffice to exceed measurement error at the individual participant-electrode level. For PECAPs, which cover the full electrode array, observing increases in η value that exceed the MDC across multiple adjacent electrodes would constitute compelling evidence of improvement in localized neural health. For polarity effects on comfort, a decrease higher than the MDC would also constitute evidence of neural health improvements, particularly when observed consistently across more than one electrode within a participant. For IPG effect variables, interpretation of directional change in relation to neural health is variable-specific, not fully established, and affected by methodological parameters (see section 4.4.2.2). However, MDC values established here provide a benchmark for observed changes beyond measurement error, with the prior expectation that, for the specific definitions used in this study, decreases in the magnitude of IPG effects on amplitude (less positive) and threshold (less negative), and increases in the magnitude of IPG effects on offset (more negative) and slope (more positive) would correspond to improvements in neural health (Vink et al., 2026). Although this study did not seek to define MCID values, the MDC estimates provide a useful starting point for interpreting longitudinal change in the examined neural-health measures.

### 4.3 Methodological considerations on assessing neural-health estimates

#### 4.3.1 Study design

Study design parameters can influence findings on neural-health estimates. In human studies, findings likely depend on participant characteristics such as age, duration of deafness, duration of implantation, and underlying auditory pathology (which itself depends on the measures used to assess it). For example, as discussed in Skidmore and He (2021), some apparently discrepant results in IPG effects might be explained by the cohort with worse neural health (with cochlear nerve deficiency diagnosed by high-resolution imaging) having a reduced number of relatively healthier neurons. This aligns with a computational study suggesting IPG offset effects depend primarily on central axon pathologies (particularly when peripheral processes are also compromised), rather than on neuron number or firing threshold (Brochier et al., 2021a); but it conflicts with guinea pig studies reporting correlations between IPG offset effects and SGN density (Ramekers et al., 2022; Ramekers et al., 2015; Ramekers et al., 2014; Schvartz-Leyzac et al., 2020a; Vink et al., 2026), although this may partly reflect differing IPG-offset definitions (see section 4.3.2). Also, it is broadly speculated that estimates from polarity and IPG effects would not always align, as the former are driven mainly by pathologies affecting the site of excitation, whereas the latter by pathologies affecting neuronal membrane integrative time constant (Guérit et al., 2018; Hughes et al., 2018). Combinations of pathologies could produce more complex patterns of effects. Reliable conclusions therefore require larger samples with well-defined inclusion criteria.

In this study, participants varied in age, duration of implantation, and other implantation characteristics, but the sample size was insufficient to explore how such factors might systematically influence neural-health estimates, limiting the generalizability of the findings. Relatedly, given the small sample size, the test-retest reliability and MDC estimates reported here should be interpreted with caution and alongside the 95% confidence intervals around the ICCs (Table 3). Also, since all participants used Advanced Bionics CIs, generalizability of results to other manufacturers’ devices is unknown. Population characteristics can also affect the number of non-existent or not meaningful data. In our dataset, there were several deactivated electrodes, and the presence of NAS required electrode substitutions or reduced current levels. These factors, alongside changes in impedances and compliance limits, contributed to a relatively high percentage of non-available data.

#### 4.3.2 Data collection and analysis

Across all three tests assessed here, testing and data collection methodological choices can affect the reliability and comparability of results. ECAP measurements are affected by parameters such as phase and IPG duration and the artifact-reduction technique, e.g., forward masking (used in this study) versus alternating polarity (Skidmore et al., 2022a; Vink et al., 2026). Forward masking further assumes complete masking of the probe response, which is only ever approximate because neural responses are probabilistic, and which requires an adequate masker level and a masker-probe interval within the refractory period of the stimulated neurons; where these conditions are not met, incomplete masking or facilitation can distort ECAP amplitudes (Boulet et al., 2016; Skidmore et al., 2024). Moreover, maximum current levels for PECAPs and IPG effects may not always reach AGF saturation, and adjustments for changes in compliance limits or loudness perception across sessions may be needed, potentially rendering results non-comparable. Selection of initial current levels used and the loudness scaling methodology for identifying comfort levels are therefore important considerations for longitudinal studies. Stimulus configuration also matters for polarity effect measures. Symmetric biphasic pulses (commonly used clinically) lack a dominant polarity, making polarity effects difficult to detect, while asymmetric pulses (such as the pseudo-monophasic pulses used here) and focused stimulation strategies more effectively reveal polarity-related differences (Guérit et al., 2018; Heshmat et al., 2021; Macherey and Cazals, 2016). Not all current-source architectures can achieve pseudo-monophasic pulses, but other asymmetries are achievable (e.g., triphasic, quadra-phasic), and polarity effects can be measured in all implant types (Carlyon et al., 2013). We also note that an issue with “symmetric” biphasic pulses used clinically is that they are never actually perfectly symmetrical because of the limited precision of the current sources. This could create small, idiosyncratic polarity effects depending on each user’s device, and is yet another reason for using asymmetric pulses here.

Focusing on IPG effects, the choice between anodic- or cathodic-leading stimuli remains unresolved, as clinical practice often favours cathodic-leading while evidence suggests anodic-leading may be more effective in humans (Skidmore et al., 2022a). Zamaninezhad et al. (2023) found that IPG effect on slope only correlated with age for cathodic- but not anodic-leading stimuli, whether the two correlations differed significantly was not reported. Results from the present study on test-retest reliability were not clearly in favor of one polarity (Table 3).

To estimate ECAP parameters for IPG effects, the approach for analysing the ECAP waveform and AGF can also significantly affect results, including selection of method for peak-picking and threshold determination, whether slope estimation is done using Boltzmann fitting, linear regression, or newer methods, and how curve offset is defined (Biesheuvel et al., 2018; Skidmore et al., 2022b). Further work on different ways of processing the acquired ECAP data could include assessing different approaches for calculating the thresholds, amplitudes, and slopes, and the application of methods for up-sampling the waveform data to allow calculation of peak latencies (Biesheuvel et al., 2018; Botros et al., 2007; Hoth et al., 2018; Skidmore and He, 2021).

Moreover, the exact definitions used to quantify IPG effects matter. For example, whether effects are assessed as absolute or proportional difference, as well as the units used for each variable, can affect results (He et al., 2026; Imsiecke et al., 2021; Yuan et al., 2022); the present study used proportional formulations for IPG amplitude (Equation 2), log-ratio for IPG threshold and for offset (Equation 4), and an absolute difference for IPG slope (Equation 3). It is important to use measures that are less affected by non-neural factors (e.g., effects being assessed as ratios rather than absolute differences). However, although using ratios is likely to more effectively remove non-neural effects (Brochier et al., 2021b), this approach may also result in negligible IPG effects on slope (Langner et al., 2021). There is also some evidence that absolute differences might correlate better with neural health (He et al., 2026; Ramekers et al., 2022; Ramekers et al., 2014; Schvartz-Leyzac et al., 2019; Takanen et al., 2024; Yuan et al., 2022), possibly because absolute differences also reflect overall ECAP amplitudes, which themselves correlate with neural health. In addition, modeling work indicates that expressing the IPG effect on slope as a ratio can remove its dependency on neural survival (Brochier et al., 2021b; Takanen et al., 2024). Also, IPG effect on amplitude and SGN density were shown to be negatively correlated when measured as a ratio (Ramekers et al., 2022; Ramekers et al., 2015), and positively correlated when measured as an absolute difference in guinea pig studies (Schvartz-Leyzac et al., 2020a; Schvartz-Leyzac et al., 2019). The IPG offset is similarly dependent on its definition, including the input-output scale used and the AGF points compared, which may contribute to discrepant findings across studies (Brochier et al., 2021b; Ramekers et al., 2022).

Furthermore, in human research, measures that do not depend on reaching saturation of the ECAP AGF are often preferred, such as assessing the IPG effect on amplitude at maximum delivered current and offset measures (Brochier et al., 2021b; Schvartz-Leyzac and Pfingst, 2018). However, these measures can still be affected by the operating point on the AGF at which delivered currents fall (e.g., being closer to threshold or to saturation).

When analyzing estimates from each of these tests, statistical methods used can also have a significant impact on study findings. For example, since all tests provide neural-health estimates for each electrode location tested, results can be presented for individual electrodes and for averages across all or subsets of electrodes. Subsets can be defined based on the apical to basal axis, or in more complex ways such as by weighting the contribution of individual electrodes based on their importance for speech recognition (Dawson et al., 2025; Schvartz-Leyzac and Pfingst, 2018; Zamaninezhad et al., 2023).

In this study, several methodological choices for IPG-effects data collection and analysis were made to address practical constraints while optimizing data quality, including the use of forward-masking artifact reduction, fixed maximum current levels constrained by comfort, semi-automated ECAP peak selection, and a sliding-window method for AGF slope calculation. Participants were also implanted with two different electrode array types (the lateral-wall HiFocus SlimJ and the pre-curved HiFocus Mid-Scala), which occupy different positions within the cochlea. Although IPG effects are largely independent of electrode-modiolus distance, these differences in array position should be considered when comparing estimates across participants. Importantly, the present work did not aim to provide an exhaustive comparison of alternative approaches for quantifying IPG-effect variables, nor to systematically evaluate how different methods influence reliability of effects, as has been explored in other studies (e.g., He et al., 2026). Consequently, alternative collection and processing strategies warrant further investigation in future work. Considering the high prevalence of non-available data arising from limited amplitude growth, approaches to reduce these occurrences are important. These could include methods for handling incomplete AGFs (e.g., principled imputation, model-based estimations) or alternative IPG variable formulations yielding stable estimates even without reaching saturation or even threshold. Such developments could help reduce missing data, improve the reliability and comparability of neural-health estimates, and enhance sensitivity to longitudinal change.

### 4.4 Potential for clinical utility to assess changes in neural health

This section discusses the potential clinical utility of PECAPs, IPG effects, and polarity effects for assessing neural health, highlighting strengths and limitations across tests. Shared strengths and limitations are summarized first, followed by test-specific evaluations.

In addition to the demonstrated high test-retest reliability, a shared strength is that all three tests are comparative measures, which can reduce the influence of non-neural factors (further details are provided per test in sections 4.4.1.1, 4.4.2.1, and 4.4.3.1). Although they require an intracochlear electrode array, and therefore cannot be used pre-operatively, they are well-suited to post-implantation applications including neural-health profiling, prognostic assessment of treatment response, longitudinal monitoring, and evaluation of therapeutic interventions.

The three tests may capture complementary aspects of neural health, an interpretation consistent with the lack of significant associations observed here. Similarly, other studies have reported no significant associations between IPG effects and polarity effects on behavioral responses (Arslan and Luo, 2022; Brochier et al., 2021a). The modeling work by Brochier et al. (2021a) further supported the interpretation that different measures index distinct aspects of neural health. Alternatively, the relationship between these variables and neural health may not be consistently monotonic (i.e., not uniformly positive or negative) but depend on additional moderating factors. Further research is needed to robustly assess these associations, ideally in larger studies with well-defined pathological subgroups.

#### 4.4.1 PECAPs

##### 4.4.1.1 Strengths and limitations

PECAPs have the advantage of assessing all electrodes with a testing duration comparable to other tests. Although direct evidence on how PECAP estimates respond to specific neural-health changes is lacking, validation work indicates that PECAPs can measure neural responsiveness across the cochlea and, in particular, identify areas of localized reduction in activity (Garcia et al., 2021). PECAPs use ECAP measurements at all probe-masker combinations and an algorithm that estimates spread of excitation (σ; reflecting non-neural effects) and neural responsiveness (η; reflecting neural health) separately for each electrode, supporting their use for neural-health profiling. In Garcia et al. (2025), PECAP estimates were less influenced by the distance between electrode arrays and SGNs than the Failure Index (FI), another test suggested to effectively remove non-neural effects (Konerding et al., 2025b). The FI measures the input (stimulation current) to output (ECAP amplitude) ratio at the upper saturation point of the AGF and represents the failure to convert delivered current into neural activation (Konerding et al., 2025b). Of note, Garcia et al. (2025) computed FI at the most comfortable level rather than at AGF (the original definition), as saturation amplitudes were not available.

One limitation of PECAPs as an outcome measure in clinical trials of therapeutics targeting neural regeneration is that it requires consistent current levels to be used for comparison of results across timepoints. At later post-implantation timepoints, the maximum allowed current may be lower than at earlier timepoints due to reduced comfort levels or compliance limits or the presence of NAS. However, this risk is generally low as, in adults, stimulation current levels at the most comfortable level and compliance limits (constrained by electrode impedances) typically either remain stable or increase after implant activation (Brotto et al., 2022; Hughes et al., 2001; Mosca et al., 2014). Recording PECAPs at the most comfortable level could help maintain consistent current levels across timepoints, providing a margin against new NAS or lower compliance limits. On the other hand, PECAPs require detectable ECAPs and thus using the highest acceptable level is more likely to produce robust results. Taken together, longitudinal PECAPs studies could use a “loud but comfortable” level, capped at a predefined maximum, expected to remain comfortable across timepoints, thereby balancing detectability against the need for stable stimulation. An important next step will be to determine how PECAP estimates obtained at a fixed stimulation level (for example, the highest level within compliance and comfort limits) compare with those obtained at constant perceived loudness, as used here. Preliminary, underpowered data indicate that neural-responsiveness estimates obtained using a fixed intra-operative stimulation level do not necessarily correspond to those obtained at a constant post-operative loudness level (Garcia, 2022). Consequently, a fixed-level protocol would require validation before it could be used to guide stimulation levels or provide reference levels for repeated longitudinal measurements. Establishing this relationship will help determine the most appropriate approach for setting stimulation levels in longitudinal protocols.

##### 4.4.1.2 Evidence on associations with neural health

The most direct evidence for the utility of PECAPs as a neural-health estimate comes from an early validation where PECAP’s neural-responsiveness estimate was found to be able to identify areas of locally reduced neural responsiveness (Garcia et al., 2021). Indirect evidence comes from observed correlations between PECAPs and other proposed neural-health measures derived from focused-stimulation thresholds (Garcia et al., 2021; Peng et al., 2025). Dawson et al. (2025) examined associations between several potential neural-health measures and speech outcomes. They found numerous significant correlations, particularly for PECAPs η averaged across the middle electrode region. However, PECAPs associations were less robust than for other measures assessed in that study (e.g., IPG offset). Also, in Peng et al. (2025), variation in PECAP estimates across the electrode array did not correlate significantly with speech perception in quiet, in contrast to variation in focused thresholds.

#### 4.4.2 IPG effects

##### 4.4.2.1 Strengths and limitations

IPG effects are largely independent of some electrode-location-related non-neural factors such as electrode-modiolus distance (Imsiecke et al., 2021; Schvartz-Leyzac et al., 2020b; Schvartz-Leyzac et al., 2025). However, IPG effects defined as linear differences remain susceptible to some non-neural factors, particularly the stimulating-recording electrode distance (Brochier et al., 2021b). Even so, expressing the IPG effect as a difference in slopes reduces, although does not fully eliminate, the influence of these factors. Importantly, the linear-slope measure remains sensitive to neural health and is among the metrics most consistently correlated with spiral ganglion neuron survival in animal studies (Ramekers et al., 2022; Ramekers et al., 2014; Schvartz-Leyzac et al., 2019; Vink et al., 2026).

Like PECAPs, IPG-effect measurements require observable ECAP waveforms (Schvartz-Leyzac et al., 2020b) and consistent current levels across timepoints. Moreover, most variables from this test require AGF analysis, which is challenging when measurements do not approach saturation or even threshold; this led to high non-availability rates in this and other studies (Arslan and Luo, 2022; Imsiecke et al., 2021). On the other hand, collecting ECAP AGF data under different IPG conditions enables analysis of multiple variables, potentially providing complementary information about underlying neural health. Testing duration (assessing three electrodes) was the shortest among the three tests, but not sufficiently short to allow testing at all electrode locations, reducing the spatial resolution of the neural-health profile obtained.

##### 4.4.2.2 Evidence on associations with neural health

Animal and human studies have linked IPG effect on amplitude to neural health. In guinea pigs, studies have found mostly significant associations (Ramekers et al., 2022; Ramekers et al., 2015; Schvartz-Leyzac et al., 2020a; Schvartz-Leyzac et al., 2019; Vink et al., 2026) but also weak associations with neural health (Ramekers et al., 2014). Across studies reporting significant findings, the direction of association appears to depend on the method used for calculating the IPG effect, particularly whether the effect was expressed as a ratio or as a linear difference. Studies in humans investigating associations between IPG effect on amplitude and speech outcomes, duration of deafness, age at implantation, and groups with different auditory nerve pathologies have reported discrepant results (He et al., 2026; He et al., 2020; Hughes et al., 2018; Imsiecke et al., 2021; Jahn and Arenberg, 2020; Schvartz-Leyzac and Pfingst, 2018). Methodological differences across these studies, including the definition of IPG effects, may explain at least some of these discrepancies (see section 4.3). Despite these inconsistencies in definitions of IPG effects, significant associations reported for age at implantation and different auditory nerve pathologies support the use of IPG effect on amplitude as a neural-health measure (He et al., 2026; He et al., 2020; Jahn and Arenberg, 2020).

IPG effect on slope has been positively correlated with SGN density in many animal studies (Ramekers et al., 2022; Ramekers et al., 2014; Schvartz-Leyzac et al., 2020a; Schvartz-Leyzac et al., 2019; Vink et al., 2026), but not others (Brochier et al., 2021b). A computational model further supported the dependency of the IPG effect on linear slope on neural survival (Takanen et al., 2024). Similarly, in humans, positive correlations with better speech perception have been reported (Schvartz-Leyzac et al., 2025; Schvartz-Leyzac and Pfingst, 2018; Zamaninezhad et al., 2023), with other studies finding no significant associations (Dawson et al., 2025; Imsiecke et al., 2021). Inverse associations between IPG effect on slope and age have also been reported, but not with duration of hearing loss (Schvartz-Leyzac et al., 2025; Zamaninezhad et al., 2023). In other studies comparing IPG effects in children with auditory nerve deficiency and those with normal-sized nerves, as well as between other clinical populations, significant differences in IPG effect on slope were found (He et al., 2026; He et al., 2020); differences in the direction of associations likely reflected heterogeneity in the underlying pathologies and methodological differences as highlighted in He et al. (2026).

For IPG offset, animal studies have consistently shown negative correlations with SGN density, i.e., more negative effects (lower current needed with longer IPGs) with higher densities (Prado-Guitierrez et al., 2006; Ramekers et al., 2022; Ramekers et al., 2015; Ramekers et al., 2014; Vink et al., 2026). In human studies, IPG offset was among the most robust CI outcome predictors in Dawson et al. (2025). However, the direction of association was opposite to that observed in animal studies, with higher currents required for shorter IPGs corresponding to poorer outcomes. Similar findings have been reported for differences across groups with deficient auditory nerve, normal nerve or other pathologies (He et al., 2026; Skidmore and He, 2021) and correlations with duration of hearing loss (Imsiecke et al., 2021) and ECAP thresholds (Sijgers et al., 2025). Moreover, other studies did not find significant association between IPG offset and place pitch sensitivity (reflecting the ability to detect pitch differences caused by changes in the location of stimulation), temporal pitch sensitivity, speech perception in quiet or noise, or pre-operative pure tone audiometry thresholds (Arslan and Luo, 2022; Imsiecke et al., 2021; Kim et al., 2010; Sijgers et al., 2025; Zamaninezhad et al., 2023).

Across studies, IPG effects on ECAP AGF linear slope or offset were also not significantly associated with the multi-pulse integration (MPI) slope, another proposed neural-health measure (Arslan and Luo, 2022; Brochier et al., 2021a; Schvartz-Leyzac et al., 2020b). Another computational study by Zhang et al. (2025) also failed to demonstrate robust associations of IPG effects on slope or offset with simulated neural-health conditions.

Findings regarding IPG effects on ECAP thresholds and wave latencies are also not entirely consistent across the literature (Dawson et al., 2025; He et al., 2026; He et al., 2020; Imsiecke et al., 2021; Jahn and Arenberg, 2020; Prado-Guitierrez et al., 2006; Ramekers et al., 2022; Ramekers et al., 2015; Ramekers et al., 2014; Schvartz-Leyzac et al., 2020a; Schvartz-Leyzac et al., 2019; Skidmore and He, 2021; Skidmore et al., 2022a; Vink et al., 2026). Effects on latency were not assessed in the present study as IPG-induced latency shifts are of comparable magnitude to the ∼18 μs sampling interval used here, and reliable estimation would have required a higher sampling rate or interpolation-based up-sampling (Ramekers et al., 2014; Skidmore and He, 2021). Nevertheless, latency-based neural-health measures may be particularly informative, as they are not dependent on response amplitude, which underpins other IPG-effect variables.

Despite some inconsistencies across studies, IPG effects are among the most well-studied experimental tests of neural health and warrant further investigation. This should include research to refine testing procedures and elucidate the mechanisms underlying observed effects and their associations with specific neural-health states.

#### 4.4.3 Polarity effects

##### 4.4.3.1 Strengths and limitations

Like the other two tests, polarity effects are comparative, measuring response differences under different conditions, which can reduce the influence of non-neural factors (Jahn and Arenberg, 2019a). The polarity-effects test offers additional advantages, including not requiring measurable ECAPs and remaining comparable even when absolute comfort levels change between visits (Undurraga et al., 2013). One disadvantage is that polarity effects are subjective psychophysical measures, with estimates depending on participants’ ability to consistently judge loudness. The structured, multi-step loudness scaling and balancing procedure minimizes variability and increases confidence in the resulting measurements. In addition, the long testing duration restricts assessment to a subset of electrodes per session, thus reducing the spatial resolution of the resulting neural-health profile, as for IPG effects. In this study, polarity effects were occasionally unavailable due to NAS. In such cases, testing of an affected electrode was discontinued and a different electrode was used instead.

##### 4.4.3.2 Evidence on associations with neural health

Although many studies have assessed the effects of stimulus polarity on electrical and behavioral responses, the underlying mechanisms are not fully understood (e.g., Guérit et al., 2018; He et al., 2025; Konerding et al., 2025a). In addition to the differential effects of stimuli with different polarities on different neural compartments, polarity effects might be shaped by other factors such as anatomical characteristics, degree of demyelination, the orientation and location of SGN peripheral processes relative to the electrodes, and characteristics of the electrical stimuli (Guérit et al., 2018; Heshmat et al., 2021; Kalkman et al., 2022; Undurraga et al., 2010; van Wieringen et al., 2008).

Several animal studies have investigated the influence of neural-health changes on electrophysiological polarity effects. Macherey and Cazals (2016) examined sisomycin-induced cochlear damage in guinea pigs (causing hair cell destruction and progressive SGN degeneration), measuring polarity effects as the current-level difference required to elicit 50% of the maximum inferior colliculus evoked potential amplitude. In many cases, the polarity effect (generally negative, i.e., lower cathodic thresholds) decreased following deafening and remained relatively stable thereafter. These findings may be specific to the aminoglycoside ototoxicity model, causing simultaneous degeneration of peripheral processes and cell bodies (Ramekers et al., 2020), to species-specific anatomical differences, such as the smaller cochlea and presence of myelinated cell bodies in guinea pigs and the different orientation of the auditory nerve fibers and length of peripheral processes between species (Rattay et al., 2001), or to differences in measurement approach. Konerding et al. (2022) further explored the relationship between polarity sensitivity and neural health in guinea pigs by introducing targeted microlesions to either SGN cell bodies (within Rosenthal’s canal) or peripheral processes (dendrites). Using biphasic stimuli and analyzing late ECAP responses, they observed polarity-dependent changes in excitability. Lesions involving the peripheral processes tended to increase the polarity effect (non-significantly), whereas cell-body lesions reduced cathodic thresholds and decreased the polarity effect. These findings may reflect, not only species-specific factors, but also the acute nature of cell-body lesioning, which could alter current pathways while leaving the central axons excitable. Konerding et al. (2025a) further investigated how neural health modulates polarity sensitivity and spike initiation sites. Their study demonstrated that chronic SGN degeneration increases the polarity effect, primarily through reduced effectiveness of cathodic-leading stimulation.

Findings from studies in humans assessing polarity effects on ECAP characteristics are also not consistent. For example, He et al. (2025) reported that polarity sensitivity of the ECAP threshold (using symmetric biphasic monopolar pulses) did not systematically differ between individuals with different expected neural survival. Hughes (2022), also using standard biphasic stimuli, identified associations between polarity effects on various ECAP characteristics and age and duration of deafness, though these relationships were inconsistent in direction and varied across implant devices. These studies are however limited by the use of symmetric pulses; because the anodic phase is generally the more effective in eliciting ECAPs (Undurraga et al., 2010), reversing the phase order would largely delay this phase rather than reveal true polarity sensitivity.

In human studies of polarity effects on behavioral responses, many findings support their potential as neural-health estimates. These include associations between polarity effects (at threshold or comfort levels) and age, duration of deafness and other potential indicators of neural health such as speech perception scores, ECAP and behavioral thresholds, pulse-rate discrimination at high rates, and spectral, temporal, and amplitude modulation detection (Carlyon et al., 2018; Dawson et al., 2025; Goehring et al., 2019; Jahn and Arenberg, 2019a; Mesnildrey et al., 2020). Across these studies, lower polarity effects corresponded with expected better neural health (e.g., younger age, shorter duration of deafness, lower response thresholds, and better performance in psychophysical hearing tasks). Conversely, many studies found no associations between polarity effects at threshold and speech perception in quiet or noise (Arslan and Luo, 2022; Jahn and Arenberg, 2019b; Mesnildrey et al., 2020). Arslan and Luo (2022) also found no association with IPG offset, MPI slope, or tests of temporal and place pitch sensitivity. These negative findings might be related to parameters of both testing (such as stimuli characteristics used and assessing polarity effects only on thresholds) and data analysis (such as averaging results across specific electrode subset and not assessing variability across the array) that affect results, but might also reflect that speech perception tasks may not optimally detect SGN degeneration.

Despite these mixed findings and remaining uncertainties in the exact underlying mechanisms, evidence across human and animal studies supports the notion that polarity effects can reflect changes in biophysical and anatomical properties of SGNs, including spike initiation site and structural integrity of different neural compartments.

#### 4.4.4 Implications for clinical development and translational use

Establishing the reliability of these measures is a prerequisite for their use in the clinical development of therapies targeting neural hearing loss. In this context, the findings inform how such measures could be deployed across different stages of therapeutic development, as detailed below.

The high test-retest reliability observed for variables across all three tests supports their candidacy as target-engagement endpoints in early-phase clinical trials. It should be noted, however, that test-retest reliability reflects measurement stability rather than sensitivity to therapy-related change, and a measure with seemingly inferior reliability could still prove more appropriate if it is more sensitive to change and the signal it captures exceeds its MDC. In populations where true neural-health change is not expected, the narrow within-unit variability and MDC values reported here serve as benchmarks for distinguishing biological effects from measurement noise in future trials. Moreover, indications that these measures capture complementary aspects of peripheral neural function support a multimodal neural-health test battery, particularly in early trials where the mechanism of action and spatial specificity of treatment effects are not yet established. Furthermore, the electrode-level resolution of these measures is especially relevant for therapies that may produce spatially heterogeneous effects along the cochlea, such as regenerative or neuroprotective interventions. Regional changes may be diluted or missed by global speech or behavioral measures, whereas electrode-specific measures could provide earlier or more spatially sensitive indications of biological response.

In addition to their role as trial endpoints, IPG effects could be applied intra-operatively, at clinically approved maximum stimulation levels, to characterize a recipient’s neural-health profile and support a more personalized approach to diagnosis, treatment, and CI programming. Realizing this will require standardized procedures and a clearer framework for the clinical interpretation of IPG results, particularly because single-timepoint measurements are currently harder to interpret than longitudinal change. Intra-operative PECAP is less practical, both because acquiring its full masker-probe matrix is time-consuming under theater time constraints and because a constant-level protocol, rather than the behavioral loudness balancing used here, would first need to be validated.

IPG effects may also be valuable for recipients who cannot provide reliable behavioral feedback about loudness, such as young children or adults with cognitive or communication impairments. Because IPG effects do not depend on loudness judgements, they could be obtained in these populations, provided protocols are developed to set safe maximum stimulation levels without behavioral input, for example using objective measures such as the electrically evoked stapedius reflex threshold (Walkowiak et al., 2025). The PECAP method as validated to date relies on behavioral loudness balancing, so its use in these populations would first require a version that does not depend on behavioral input.

To realize the full potential of these tests, future work should focus on optimizing their implementation to facilitate clinical adoption and broader research use. In particular, testing procedures can be streamlined to reduce assessor and participant burden (for example testing duration) and to refine protocols for improved data quality and clinical-trial applicability. For the IPG-effect test in particular, this could include simplifying the procedure, for example by using a single polarity or an alternating-polarity paradigm. Such efforts are already underway, including refinement of methods for an upcoming study in acutely implanted participants, with planned integration of PECAPs and IPG effects into a single software application and improvements to the user-friendliness of the polarity effects tool. Evaluation of these measures in acutely implanted participants will further inform clinical applicability and establish reference values for interpreting longitudinal changes in neural health following implantation. In parallel, continued protocol refinement and standardization will enhance reliability and comparability across centers, devices, and time points, supporting wider translation and scalable use in clinical studies.

Beyond early-phase development, reproducible neural-health measures have the potential to play a supportive role for regulatory and reimbursement decision-making as complementary, mechanistically informed endpoints. While such measures are not intended to replace patient-centered functional outcomes, they could act as informative co-endpoints by providing direct evidence of target engagement and physiological change, thereby strengthening interpretation of equivocal, heterogeneous, or delayed functional responses. Realizing this potential will involve establishing clinically meaningful thresholds of change, clarifying links between neural-health improvements and downstream functional outcomes, and demonstrating robustness across multicentre and real-world settings. The present study contributes an important foundation for this work by defining the reliability and measurement limits within which future translational, regulatory, and health-economic questions can be confidently addressed.

## 5 Conclusion and future directions

This study demonstrates that PECAPs, IPG effects, and polarity effects can be obtained reliably and repeatably when performed by appropriately trained clinical audiologists. Each test exhibits distinct strengths and limitations, with largely independent estimates, supporting their complementary use as part of a combined neural-health assessment. Whilst it remains to be fully established whether specific measures are preferentially sensitive to particular neural pathologies such as loss of peripheral processes, central axon demyelination, or complete loss of neurons (Brochier et al., 2021a), the observed independence of estimates is consistent with the possibility that they probe different aspects of neural health. Despite discrepancies in the existing literature and incomplete understanding of underlying mechanisms, the present findings support the feasibility and value of incorporating these measures in clinical-trial settings. In particular, the combined application of PECAPs, IPG effects, and polarity effects in clinical trials targeting neural health may improve detection of treatment-related physiological change and provide mechanistic context alongside functional outcomes. In this way, the study represents an important step toward evaluating the translational relevance of these neural health measures, including their potential role as supportive co-endpoints in regulatory and reimbursement frameworks.

Continued improvement of neural-health measures nevertheless remains important. For the tests assessed here, further methodological refinements are expected to enhance efficiency, usability, and measurement quality. In parallel, a range of additional candidate measures may capture complementary aspects of neural function, including electrically-evoked ABRs (Bayrak et al., 2019; Chen et al., 2025; Gibson et al., 2009) and other advanced ECAP-based measurements such as focused thresholds, refractory time, the failure index, and inter-pulse interval effects on ECAPs (Brochier et al., 2021a; Chen et al., 2025; Dawson et al., 2025; Konerding et al., 2025b; Skidmore et al., 2022a). As evidence accumulates, systematic reviews will be important for identifying the most informative combination of complementary assessments and for guiding standardization of testing and analysis. This evidence will also enable optimization of the battery itself, by defining a core subset of complementary measures to reduce testing burden and incorporating emerging measures, where appropriate, for specific clinical or research applications.

In summary, the test battery assessed here provides a robust and reproducible approach for evaluating neural health in CI users. Ongoing refinement, validation, and expansion of these methods, alongside longitudinal and multicentre studies, will further support their clinical utility and advance understanding of neural-health changes in the context of therapies targeting neural hearing loss.

## Data Availability

The datasets presented in this article are not readily available because the datasets include sensitive clinical and electrophysiological information from cochlear implant users that may be identifiable from such data. These data contain high dimensional measurements at the individual electrode level; therefore, full anonymization cannot be guaranteed without compromising data integrity. In accordance with ethical restrictions imposed by the UK HRA, the East Midlands - Nottingham 2 Research Ethics Committee, and the terms of participant consent, the raw data cannot be made openly available. Data access is therefore restricted to protect participant confidentiality. Qualified researchers may request controlled access to the data by contacting the corresponding author, subject to institutional approval and appropriate data-sharing agreements. Requests to access the datasets should be directed to Eleni Genitsaridi, eleni.genitsaridi@rinri-therapeutics.com.
