# Supplementary material for: Establishing reliable neural health measures in adult cochlear implant users
Source: Front Neurosci. 2026 Aug 13;20:1890411. doi: 10.3389/fnins.2026.1890411 (PMC13518592; doi:10.3389/fnins.2026.1890411)
Supplement: Supplementary file 1 [file Data_Sheet_1.docx]

Supplementary Material

# 1 Supplementary Figures and Tables

## 1.1 Supplementary Figures

**Supplementary Figure 1.** Example of calculation of IPG offset effect. Example from measurements obtained from participant 4, apical electrode (e3), session 2 using cathodic-leading stimuli. Both amplitude growth functions are shown on logarithmic input-output axes (stimulus current in dB re 1 μA; ECAP amplitude in dB re 1 μV). Blue lines (right) correspond to the 0-μs IPG stimuli and orange lines (left) to the 30-μs IPG stimuli. The y-axis start marks the 10 μV noise floor, and the two horizontal lines mark the 70% and 90% limits of the amplitude range (defined from the noise floor to the smaller of the two curve maxima), between which the offset is evaluated. The offset is the mean horizontal separation (difference in stimulus current, in dB) between the two functions at matched ECAP amplitude, averaged over this band on a 0.1 dB grid. In this example, averaged across the band, the IPG offset was −2.38 dB.

**Supplementary Figure 2.** Impedances measured before and after each testing session.

## 1.2 Supplementary Tables

Supplementary Table 1. Descriptive statistics for all reported variables

|  | **Count** | **Non-available (%)** | **Mean** | **SD** | **Min** | **Max** |
| --- | --- | --- | --- | --- | --- | --- |
| PECAPs η (rescaled) | 480 | 63 (13.13) | 0.137 | 0.174 | 0.002 | 0.785 |
| PECAPs η (scaled) | 480 | 63 (13.13) | 0.247 | 0.222 | 0.006 | 0.918 |
| IPG threshold (CL) (dB) | 90 | 27 (30.00) | -1.372 | 1.133 | -2.667 | 2.239 |
| IPG threshold (AL) (dB) | 90 | 27 (30.00) | -1.292 | 0.883 | -2.778 | 1.719 |
| IPG amplitude (CL) | 90 | 27 (30.00) | 0.322 | 0.093 | 0.141 | 0.551 |
| IPG amplitude (AL) | 90 | 27 (30.00) | 0.346 | 0.102 | 0.155 | 0.520 |
| IPG slope (CL) (μV/μA) | 90 | 27 (30.00) | 0.298 | 0.258 | -0.142 | 1.074 |
| IPG slope (AL) (μV/μA) | 90 | 27 (30.00) | 0.354 | 0.208 | -0.041 | 0.784 |
| IPG offset (CL) (dB) | 90 | 27 (30.00) | -1.953 | 0.517 | -2.802 | 0.024 |
| IPG offset (AL) (dB) | 90 | 27 (30.00) | -1.869 | 0.282 | -2.443 | -0.880 |
| Polarity threshold (dB) | 90 | 7 (7.78) | 1.013 | 1.814 | -5.105 | 5.137 |
| Polarity comfort (dB) | 90 | 8 (8.89) | 2.391 | 1.612 | -0.200 | 5.400 |
| ECAP thresholds (cu) | 480 | 20 (4.17) | 161.332 | 57.487 | 11.000 | 250.000 |
| BKB SNR (dB) | 6 | 0 (0.00) | 8.917 | 7.050 | 0.200 | 20.200 |
| AB quiet words (%) | 6 | 0 (0.00) | 57.778 | 15.197 | 33.333 | 70.000 |
| AB noise words (%) | 6 | 0 (0.00) | 18.889 | 10.179 | 10.000 | 36.667 |
| Impedances (kOhm) | 960 | 48 (5.00) | 5.411 | 2.536 | 2.515 | 15.372 |

Counts for session × participant × electrode. All variables were assessed once at each session, except impedances which were assessed twice per session, and speech perception variables which were assessed in one session only. Three electrodes were assessed for IPG effects and polarity effects, and all 16 electrodes for PECAPs, ECAP thresholds and impedances.

For PECAPs data from e15 and e16 for participant 1, and for e1 for participant 6 were not collected (this is because the electrodes were deactivated in the participants’ clinical programmes). For participant 6, e15 was also deactivated but PECAPs estimates could be generated by interpolating measurement values for e15 using e14 and e16 values. Also, for participant 3, all measurements from session 1 that were done at a higher level (being non-comparable to data from other visits) were removed (lower levels used in subsequent sessions due to increase in impedance leading to reduction in compliance limits). Also, measurements from participant 5 from sessions 1 and 2 were done at a higher level and were removed (lower levels used in subsequent sessions due to NAS).

For IPG effects, most non-available data were because of insufficient amplitude growth at increasing currents (including all data for participant 6). Also, as for PECAPs, data from participant 3, session 1 were collected at a higher level than for following sessions and were removed from analysis as non-comparable.

For polarity effects, for participant 1 session 2, polarity threshold was not assessed as the audiologist performing the test (for the first time) had misinterpreted instructions. For participant 4, polarity testing was not done during session 1 due to time constraints. Additional non-existent data from participants 5 and 6 were due to NAS during testing. For participant 6, sessions 3 to 5, e12 was tested instead of e13, because participant was experiencing NAS at e13. Measurements available for either e12 or e13 for this participant were not counted as non-available since they were used in at least one analysis.

The non-available data for ECAP thresholds were from e15 and e16 from participant 1 and e1 and e15 from participant 6; these electrodes were deactivated.

The missing values for impedances were from 3 sessions when impedances were not repeated.

AL: Anodic-leading; CL: Cathodic-leading; NAS: non-auditory sensations.

Supplementary Table 2: Average values across sessions for each participant-electrode unit

| **Participant** | **Electrode** | **Age** | **BKB SNR** | **AB quiet words** | **AB noise words** | **PECAPs η (rescaled)** | **Polarity comfort** | **IPG slope (AL)** | **ECAP threshold** |
| --- | --- | --- | --- | --- | --- | --- | --- | --- | --- |
| **PT 1** | **3** | 72.1 | 20.2 | 40.0 | 10.0 | 0.40 | 0.78 | 0.47 | 164.00 |
| **PT 1** | **8** |  |  |  |  | 0.10 | 1.08 | 0.22 | 240.20 |
| **PT 1** | **13** |  |  |  |  | 0.39 | 1.84 | 0.35 | 136.23 |
| **PT 2** | **3** | 25.3 | 5.7 | 66.7 | 20.0 | 0.05 | 2.08 | 0.49 | 176.33 |
| **PT 2** | **8** |  |  |  |  | 0.52 | 5.18 | 0.67 | 170.28 |
| **PT 2** | **13** |  |  |  |  | 0.68 | 5.10 | 0.44 | 130.15 |
| **PT 3** | **3** | 31.5 | 0.2 | 66.7 | 26.7 | 0.01 | 2.24 | 0.55 | 113.78 |
| **PT 3** | **8** |  |  |  |  | 0.01 | 0.92 | 0.71 | 194.85 |
| **PT 3** | **13** |  |  |  |  | 0.20 | 3.36 | 0.18 | 117.25 |
| **PT 4** | **3** | 52.2 | 9.7 | 70.0 | 10.0 | 0.05 | 3.95 | 0.10 | 158.53 |
| **PT 4** | **8** |  |  |  |  | 0.20 | 4.58 | 0.15 | 155.43 |
| **PT 4** | **13** |  |  |  |  | 0.35 | 1.58 | 0.12 | 56.30 |
| **PT 5** | **3** | 57.8 | 2.5 | 70.0 | 36.7 | 0.17 | 2.16 | 0.64 | 141.18 |
| **PT 5** | **8** |  |  |  |  | 0.19 | 4.60 | 0.22 | 186.48 |
| **PT 5** | **13** |  |  |  |  | 0.03 | 2.86 | 0.21 | 176.40 |
| **PT 6** | **3** | 68.9 | 15.2 | 33.3 | 10.0 | 0.62 | 1.08 |  | 250.00 |
| **PT 6** | **8** |  |  |  |  | 0.07 | 0.46 |  | 62.58 |
| **PT 6** | **13** |  |  |  |  | 0.08 | 0.10 |  | 131.38 |
| **PT 6** | **12** |  |  |  |  | 0.08 | 0.60 |  | 250.00 |
